# Metabolite-based dietary supplementation in human type 1 diabetes is associated with microbiota and immune modulation

**DOI:** 10.1186/s40168-021-01193-9

**Published:** 2022-01-19

**Authors:** Kirstine J. Bell, Sonia Saad, Bree J. Tillett, Helen M. McGuire, Sara Bordbar, Yu Anne Yap, Long T. Nguyen, Marc R. Wilkins, Susan Corley, Shannon Brodie, Sussan Duong, Courtney J. Wright, Stephen Twigg, Barbara Fazekas de St Groth, Leonard C. Harrison, Charles R. Mackay, Esteban N. Gurzov, Emma E. Hamilton-Williams, Eliana Mariño

**Affiliations:** 1 Charles Perkins Centre, University of Sydney, Camperdown, Sydney, New South Wales 2050 Australia; 2 Faculty of Medicine and Health, Sydney Medical School, University of Sydney, Sydney, New South Wales Australia; 3 Kolling Institute of Medical Research, Royal North Shore Hospital, Sydney Medical School, University of Sydney, St Leonards, Sydney, New South Wales Australia; 4 The University of Queensland Diamantina Institute, The University of Queensland, Woolloongabba, Brisbane, Queensland 4102 Australia; 5 Discipline of Pathology, Faculty of Medicine and Health, The University of Sydney, Camperdown, Sydney, New South Wales 2050 Australia; 6 Ramaciotti Facility for Human Systems Biology, The University of Sydney, Camperdown, Sydney, New South Wales 2050 Australia; 7 Infection and Immunity Program, Biomedicine Discovery Institute, Department of Biochemistry, Monash University, Melbourne, Victoria 3800 Australia; 8 Systems Biology Initiative, School of Biotechnology and Biomolecular Sciences, University of New South Wales, Sydney, New South Wales 2052 Australia; 9 Walter and Eliza Hall Institute of Medical Research, Parkville, Melbourne, Victoria 3052 Australia; 10 Department of Medical Biology, University of Melbourne, Melbourne, Victoria 3010 Australia; 11 Signal Transduction and Metabolism Laboratory, Université libre de Bruxelles, 1070 Brussels, Belgium

**Keywords:** Type 1 diabetes, SCFAs, Microbiota, Immune regulation, Dietary-metabolites, Autoimmunity

## Abstract

**Background:**

Short-chain fatty acids (SCFAs) produced by the gut microbiota have beneficial anti-inflammatory and gut homeostasis effects and prevent type 1 diabetes (T1D) in mice. Reduced SCFA production indicates a loss of beneficial bacteria, commonly associated with chronic autoimmune and inflammatory diseases, including T1D and type 2 diabetes. Here, we addressed whether a metabolite-based dietary supplement has an impact on humans with T1D. We conducted a single-arm pilot-and-feasibility trial with high-amylose maize-resistant starch modified with acetate and butyrate (HAMSAB) to assess safety, while monitoring changes in the gut microbiota in alignment with modulation of the immune system status.

**Results:**

HAMSAB supplement was administered for 6 weeks with follow-up at 12 weeks in adults with long-standing T1D. Increased concentrations of SCFA acetate, propionate, and butyrate in stools and plasma were in concert with a shift in the composition and function of the gut microbiota. While glucose control and insulin requirements did not change, subjects with the highest SCFA concentrations exhibited the best glycemic control. *Bifidobacterium longum*, *Bifidobacterium adolescentis*, and vitamin B7 production correlated with lower HbA1c and basal insulin requirements. Circulating B and T cells developed a more regulatory phenotype post-intervention.

**Conclusion:**

Changes in gut microbiota composition, function, and immune profile following 6 weeks of HAMSAB supplementation were associated with increased SCFAs in stools and plasma. The persistence of these effects suggests that targeting dietary SCFAs may be a mechanism to alter immune profiles, promote immune tolerance, and improve glycemic control for the treatment of T1D.

**Trial registration:**

*ACTRN12618001391268. Registered 20 August 2018,* *https://www.anzctr.org.au/Trial/Registration/TrialReview.aspx?id=375792*

Video Abstract

**Supplementary Information:**

The online version contains supplementary material available at 10.1186/s40168-021-01193-9.

## Background

Short-chain fatty acids (SCFAs) are metabolites produced by the gut microbiota, that greatly affect human health and disease [1]. Mostly produced from fermentation of non-digestible dietary carbohydrates, SCFAs, primarily acetate, propionate, and butyrate, are anti-inflammatory and critical for maintaining gut homeostasis as well as playing a role in host energy metabolism [2]. Reduced SCFA production is an indicator of a loss of beneficial bacteria (dysbiosis), which is commonly associated with chronic autoimmune and inflammatory diseases including type 1 diabetes (T1D) and type 2 diabetes [3, 4]. Indeed, studies in mice and humans have shown that a deficiency in production of SCFAs by the gut microbiota is associated with the development of T1D, starting well before clinical diagnosis [5–8], which may be linked to altered gut homeostasis [9–11]. Insufficient intake of dietary fiber, or a gut microbiota that is poor at SCFA production may underpin the increased incidence of T1D and many other Western diseases [1, 2]. Thus, a microbiota-targeted dietary intervention that tackles the underlying functional dysbiosis (i.e., deficiency of SCFAs and altered microbiota function) may have great potential in humans to prevent or treat T1D, as it does in autoimmune diabetes-prone nonobese diabetic (NOD) mice [5].

Clinical studies have begun to show the potential of modulating the microbiota composition via the use of prebiotics, probiotics, and fecal transplantation as an alternative approach to treat inflammatory diseases [12]. Most microbiota-based therapies identify bacterial communities or pathways associated with disease and then aim to restore specific health-associated species, an approach with inherent caveats. For example, the wide inter-individual variability in the gut microbiota associated with diseases such as T1D [3, 7] and key factors such as diet and host genetics, which affect microbiota colonization and adaptation, are all barriers to current investigative approaches [13]. Post-biotic targets (metabolites produced by the gut microbiota) have recently emerged as a novel alternative to promote health and circumvent these barriers [14]. We have developed a high-SCFA–yielding dietary supplement that ameliorates gut infection, improves chemotherapy efficacy, and protects mice against T1D via regulating the immune system [5, 10, 15]. The SCFA-based supplement is a type 2–resistant starch consisting of a high-amylose (70%) maize starch (HAMS) that has been modified by bonding the acetate and butyrate (HAMSAB). It is resistant to digestion in the upper gastrointestinal tract, delivers a very high yield of SCFAs in the colon, and is a powerful tool to assess the effects of SCFAs on intestinal biology [5, 10, 15, 16]. The HAMSAB diet prevented beta-cell destruction by T cells and protected against T1D in 90% of NOD mice. Protection against T1D was associated with microbiota composition changes. The mechanism behind this SCFA-induced T1D protection involved synergistic effects of acetate and butyrate; expansion of regulatory T cells (Treg) was butyrate dependent, and a decrease in pathogenic B cells and CD4^+^ and CD8^+^ T cells was acetate dependent [5]. Therefore, this dietary intervention targets microbiota–host interactions, nutrition, and phylogeny.

Several human studies support the positive effects of increasing total fiber consumption in the form of high-amylose maize-resistant starch (HAMS) or butyrate-yielding HAMS (HAMSB) supplementation alone in glycemic control [17, 18] or gastrointestinal conditions [19–21]. We report the first interventional study in humans with T1D to determine the effects of delivering a combination of dietary acetate and butyrate on the immune system via the gut microbiota. We therefore, conducted a single-arm pilot-and-feasibility study using HAMSAB administered for 6 weeks with follow-up at 12 weeks in adults with long-standing T1D.

## Results

### Clinical characteristics and protocol adherence

**Fig. 1:**
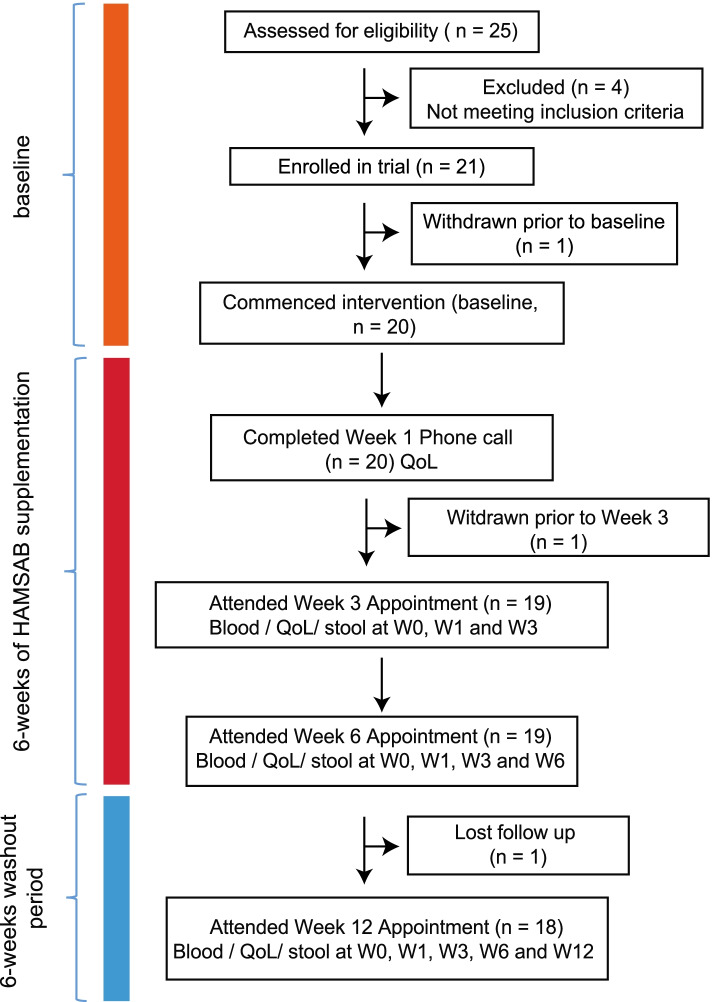
Experimental study in patients with T1D. Schematic diagram illustrating the study design and selection procedure for the individuals enrolled in the study

The primary outcome of our study was to determine the safety and feasibility of delivery of a HAMSAB dietary supplement in adults with T1D, while the secondary outcomes included determining the effects of the supplement on glycemic control, the gut microbiota, and immune cell function. To assess these aims, a total of 25 subjects between 18 and 45 years of age, diagnosed with T1D for at least 6 months and had HbA1c ≤ 8.5% were screened. Among this group, 20 subjects commenced HAMSAB supplementation (Fig. 1). Participants consumed 40 g/day of the HAMSAB supplement (20 g in the morning and at night) for 6 weeks with follow-up at 12 weeks. The number of subjects enrolled and the dose of 40 g per day was based on a power calculation from a previous study in which 20–40 g of HAMSB per day was administered resulting in increased butyrate concentrations in stool and altered microbial composition [22]. Participants had been diagnosed with T1D for a mean of 14 ± 12 years, 60% used multiple daily injection insulin therapy and mean HbA1c at baseline was 7.1 ± 0.6% (54 ± 7 mmol/mol) (Table 1). Two subjects withdrew, and one needed to halve the dose from week 3 onwards due to gastrointestinal side effects, as per protocol. Eighteen (90%) of 20 subjects starting the HAMSAB supplement remained in the trial and on the full dose for the entire 6 weeks (Fig. 1). Participants consumed 94 ± 10% of all doses based on the participants’ self-reported logbooks and 95 ± 8% based on returned unused supplement thus, indicating high compliance with the intervention and meeting the primary outcome of study feasibility.

**Table 1:** Baseline and follow-up characteristics

	Visit 1 (baseline)	Visit 2 (week 3)	Visit 3 (week 6)	Visit 4 (week 12)
**Age (median years, min–max))**	36.5 (18–45)			
**Male,** ***n*/total (female %)**	9/20 (55%)			
**Diabetes duration years mean and (IQR)**	14 ± 12 11 (18)			
**Insulin therapy mode (% MDI)**	60%			
**Weight (kg)**	76.2 ± 13.9 (*n* = 20)	75.8 ± 13.8 (*n* = 19)	77.3 ± 13.4 (*n* = 19)	77.1 ± 14.0 (*n* = 18)
**Waist circumference (cm)**	85.8 ± 11.5 (*n* = 20)	84.7 ± 11.8 (*n* = 19)	86.2 ± 11.6 (*n* = 19)	85.4 ± 11.6 (*n* = 18)
**HbA1c (% [mmol/mol])**	7.1 ± 0.6 [54 ± 7] (*n* = 18)		7.0 ± 0.7 [53 ± 8] (*n* = 18) (W0 vs W6, *P* = 0.33)	7.3 ± 0.8 [56 ± 8] (*n* = 18) (W0 vs W12, *P* = 0.06) (W6 vs W12, *P* < 0.001)
**Daily average glucose (mmol/L)**	8.8 ± 1.4 (*n* = 19)	9.5 ± 1.9 (*n* = 17)	9.6 ± 1.4 (*n* = 16)	
**Time in target range** **(3.9–10.0 mmol/L) (%)**	58.0 ± 15.0 (*n* = 19)	51.0 ± 20.0 (*n* = 17)	51.0 ± 15.0 (*n* = 16)	
**Time below target range (< 3.9 mmol/L) (%)**	8.0 ± 6.0 (*n* = 19)	7.0 ± 4.0 (*n* =17)	7.0 ± 6.0 (*n* = 16)	
**Time above target range** **(> 10.0 mmol/L) (%)**	34.0 ± 16.0 (*n* = 19)	42.0 ± 21.0 (*n* =17)	41.0 ± 15.0 (*n* = 16)	
**Standard deviation of CGM blood glucose values (mmol/L)**	3.5 ± 0.8 (*n* = 18)	3.9 ± 1.0 (*n* = 17)	4.0 ± 1.0 (*n* = 16)	
**Insulin total daily dose (units)**	43.6 ± 15.5 (*n* = 20)	45.0 ± 15.0 (*n* = 19)	43.8 ± 15.6 (*n* = 18)	47.9 ± 17.9 (*n* = 18)
**Basal insulin dose (%)**	22.4 ± 7.6 (*n* = 20)	22.8 ± 7.0 (*n* = 19)	21.5 ± 7.0 (*n* = 18)	22.8 ± 7.0 (*n* = 18)
**Lymphocytes (×109/L)**	1.7 ± 0.4 (*n* = 20)	1.8 ± 0.6 (*n* = 19)	1.7 ± 0.5 (*n* = 17)	

Data are expressed as means ± standard deviation. *P* values were calculated using a GEE model for each outcome measure. Where the main effect of time was shown significant, repeated measures *t* tests were used to assess the estimated change in the outcomes over time. Diabetes duration also expressed in years as mean and (IQR). *IQR*= inter-quartile range

Mean HbA1c remained unchanged from baseline (W0) at both week 6 (W6) and week 12 (W12) (Table 1). Based on the continuous glucose monitoring (CGM) data, the mean daily average blood glucose and the mean standard deviation of CGM blood glucose values were not significantly different across the study (Table 1). Insulin dosing also remained stable throughout the study with no difference in the proportion of basal versus bolus insulin (Table 1). Only 31.5% (6/19) participants had detectable fasting plasma C-peptide measured by multiplex at the start of the study, the concentration of which did not change (data not shown). However, plasma C-peptide was not detected at fasting or at any timepoint during the 2-h Mixed Meal Tolerance Test (MMTT; data not shown). These data showed that the supplement was safe with regards to diabetes management.

The primary outcome of tolerability measured by gastrointestinal symptoms in relation to HAMSAB intake remained consistent or improved throughout the trial (Table S1A). Significantly higher symptom scores, indicating greater quality of life, were reported in W1 and W12 compared with baseline (Table S1A). Consistent with our data, other resistant starch intervention trials have reported that participants with no functional gastrointestinal disorders can tolerate high daily doses of resistant starch up to 50 g per day [18, 22–24]. No changes in nutritional intake, assessed by 3-day food diaries, were observed between W0 and W6 (Table S1B). Weight and waist circumference remained stable throughout the trial (Table 1). Nineteen participants completed the supplement acceptability survey at W3 and W6. The majority of participants found the supplement acceptable through the length of the treatment. Twenty-six adverse events were reported during the trial, but approximately half (12/26) were unrelated to either the supplement or study protocol. The remainder related to minor gastrointestinal side effects. No serious adverse events and no toxicity events were associated with the HAMSAB supplement dose or time on supplementation. Thus, data showed a dose of 40 g of HAMSAB supplement was safe and tolerable in adults with T1D, and the study design was feasible, meeting the primary outcome of the study.

### HAMSAB supplementation is followed by increased SCFAs in stool and circulation

**Fig. 2:**
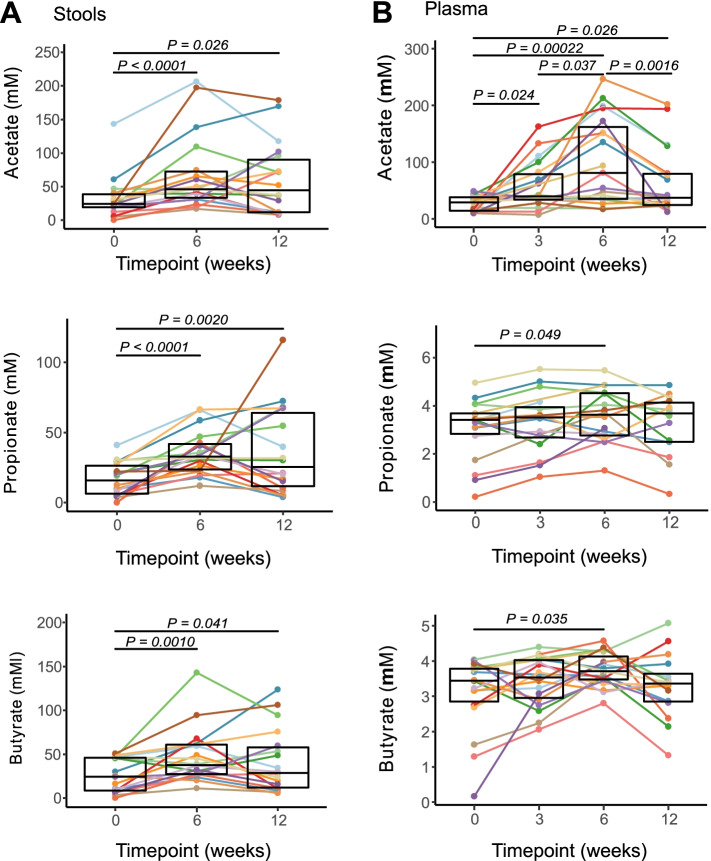
Increased concentration of short-chain fatty acids in stool and plasma following HAMSAB supplementation. **A** Acetate, propionate, and butyrate concentrations in stool (mM) and **B** plasma (μM). Overall significance determined by GEE and pairwise differences between timepoints by estimated marginal means and include a Tukey adjustment for multiple corrections. Colors indicate individual subjects. Box plots show mean and upper and lower quartile ranges

Similar to mice, several studies have reported that humans with T1D have reduced concentrations of stool and plasma SCFAs [5, 6, 8]. Consumption of the HAMSAB supplement over 6 weeks was followed by increased acetate, butyrate, and propionate concentrations in both stool and plasma (secondary outcome) (Fig. 2). In the stool, the majority (77.7%) of subjects had > 2-fold increase in acetate and propionate, and 61% subjects had > 2-fold increase in butyrate concentration at W6 (Fig. 2A). Following the 6-week washout period, over 50% of subjects continued to have > 2-fold higher concentration of SCFAs in the stool compared with baseline. In plasma, acetate concentrations started to rise significantly after 3 weeks on HAMSAB supplementation reaching a maximum peak at W6 (58% subjects > 2-fold plasma acetate increased, Fig. 2B). To a lesser but also significant degree, propionate and butyrate increased in the plasma at W6. At W12, at the completion of the 6-week washout period, 8/19 subjects continued to maintain elevated concentrations of acetate in the plasma, but not propionate or butyrate. Thus, an increased SCFA availability in both the large intestine and circulation occurred after HAMSAB supplementation.

### HAMSAB delivery is linked with changes in the gut microbiota

**Fig. 3:**
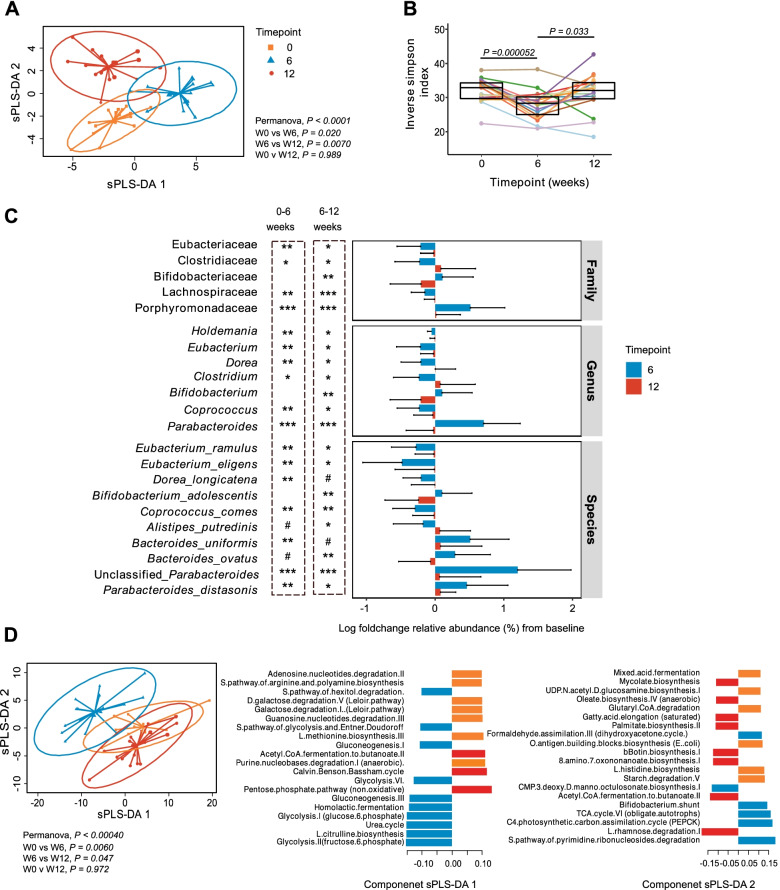
Composition and function of the gut microbiome is altered following HAMSAB supplementation. **A** Multivariate sPLS-DA comparing microbial species present at each timepoint. Significance determined by PERMANOVA. Loadings shown in Fig S1. **B** Alpha diversity measured by inverse Simpson index. Overall significance determined by GEE and pairwise differences between timepoints by estimated marginal means. Colors indicate individual participants. Box plots show mean and upper and lower quartile ranges. **C** Mean log foldchange in relative abundance from baseline at W6 and W12, grouped by taxonomic classification. Asterix represents GEE significance of changes in abundance from baseline. Error bars represent standard deviation. # adjusted *P* < 0.1, *adjusted *P* < 0.1–0.05, **adjusted *P* < 0.01, ***adjusted *P* < 0.001. **D** Multivariate sPLS-DA comparing microbial pathways present at each timepoint and plot loadings indicating the contribution of each bacterial function to the variance. Color corresponds to the timepoint

High-depth metagenomic sequencing of the stool microbiota found that taxonomic composition of the microbiome (secondary outcome) at W6 differed from baseline and W12, which were more similar in composition (Fig. 3A). Multivariate sPLS-DA analyses determined the changes in the microbiome at W6 were associated with unclassified members of the genus *Parabacteroides, Parabacteroides distasonis*, and *Parabacteroides merdae* as well as *Bacteroides ovatus, Bifidobacterium adolescentis*, and* Dialister invisus* (Fig. 3A and Fig. S1A). In line with this change, alpha diversity was reduced at W6 on completion of the supplement, returning to similar diversity as baseline at W12 (Fig. 3B). Univariate analyses also identified significant increases in *Bacteroides uniformis,* unclassified *Parabacteroides,* and *P. distasonis* from baseline to W6 (Fig. 3C). Meanwhile, *Eubacterium ramulus, Eubacterium eligens*, and *Coprococcus comes*, which are known to feed on other carbohydrate sources such as pectin and flavonoids [25–27], were all decreased from baseline to W6 (Fig. 3C). *B. adolescentis* and *B. ovatus* marginally increased at W6 and then dropped back at W12. *Alistipes putredinis* showed the opposite trend, marginally decreasing at W6 and then significantly increasing at W12. Together, these data show a profound change in the composition of the gut microbiota following the HAMSAB diet, likely indicating expansion of bacteria able to use the delivered supplement.

Next, a change in the metabolic pathways used by microbiota was investigated. The microbiome functions identified by metagenomic sequencing changed substantially at W6, but this was not sustained at follow-up (Fig. 3D). The pathways driving the changes at W6 included multiple carbohydrate energy production pathways including glycolysis and gluconeogenesis pathways, fermentation pathways such as homolactic fermentation and bifido-shunt, and amino acid biosynthesis pathways (urea cycle and L citrulline biosynthesis) (Fig. 3D). Univariate comparisons identified 46 individual pathways that differed in abundance at W6 (Table S2). These were categorized within 15 superclasses. Glycolysis pathways, amino acid biosynthesis pathways (urea cycle and L-citrulline biosynthesis) and the biosynthesis of cofactors and vitamins (B2 (flavin), B6 (pyridoxal), B7 (biotin), and B9 (folate)) all increased from baseline to W6, but this increase was not sustained at W12 follow-up. In contrast, acetyl CoA fermentation to butanoate decreased at W6 and then increased again at W12, suggesting a switch occurs in SCFA production from starch utilization via glycolysis while on the HAMSAB supplementation, returning to SCFA production via fermentation at follow-up. Amino acid, nucleotide, and vitamin biosynthesis can all be fueled by acetate utilization [10, 28]. These data indicate that HAMSAB supplementation was associated with a substantial shift in both the composition and function of the gut microbiota.

### HAMSAB supplementation was followed by increased circulating marginal zone B cells with a lower activation status

**Fig. 4:**
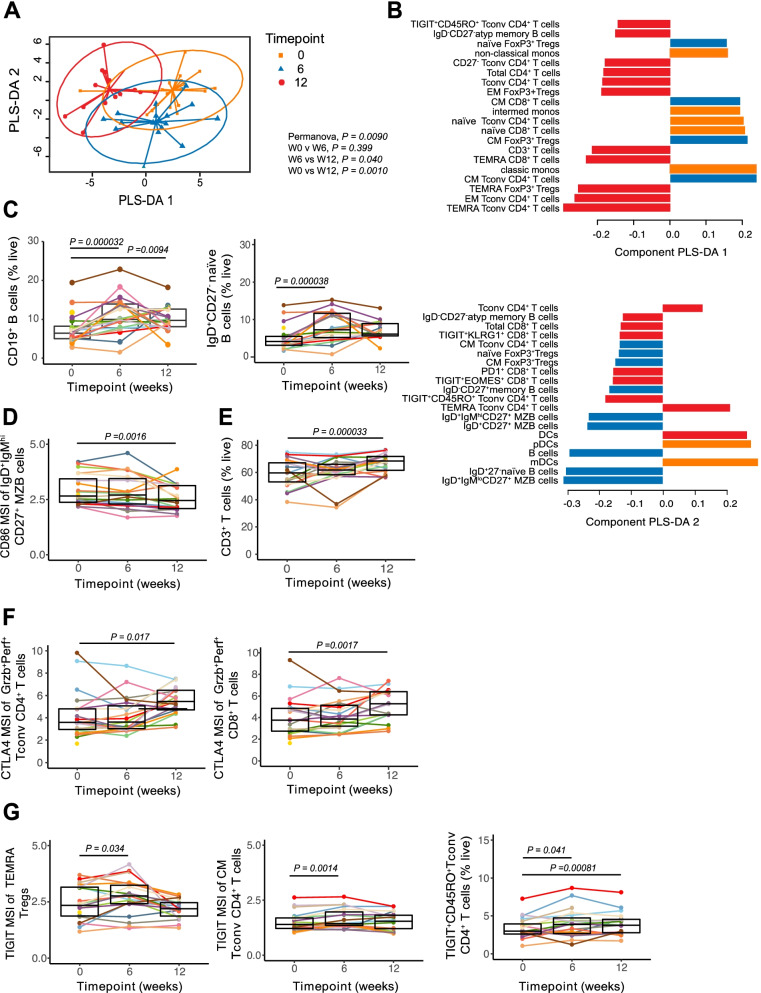
HAMSAB supplementation is accompanied by modulation of the immune system at W6 and W12 follow-up. A Multivariate PLS-DA comparing the proportions of major immune populations assessed by mass cytometry within total live cells. Significance determined by PERMANOVA. **B** PLS-DA plot loadings indicating the contribution of each immune population. **C** Proportions of total CD19^+^ B cells and IgD^+^CD27^-^ naïve B cells within live cells. **D** IgD^+^IgM^hi^CD27^+^ MZ B cells expressing CD86 (mean geometric signal intensity, MSI). **E** CD3^+^ T cell % within live cells. **F** CTLA4 expression (MSI) on granzyme B^+^ perforin^+^ (Grzb^+^Perf^+^) Tconv CD4^+^ T cells and Grzb^+^Perf^+^ CD8^+^ T cells. **G** TIGIT expression (MSI) on TEMRA Tregs, CM Tconv CD4^+^ T cells and % TIGIT^+^CD45RO^+^ Tconv within live cells. Colored dots and lines represent each subject. Box plots show mean and upper and lower quartile ranges. Significance determined by GEE. Adjusted *P* values are (6W vs W0) or (12W vs W0). Gating strategy shown in Fig. S2

Previously, we reported that increasing SCFAs in NOD mice modulated autoimmune B and T cell responses in T1D, and this was associated with protection against disease [5]. Thus, in a mechanistic analysis using mass cytometry, we evaluated here whether HAMSAB supplementation changed immune cell subsets within peripheral blood mononuclear cells (PBMC) (secondary outcome). A multivariate PLS-DA visualization distinguished changes in the total proportion of multiple cell types across time (Fig. 4A). The overall changes in PBMCs were mainly seen at W12 compared with either baseline or W6 (Fig. 4A). Changes at W12 were related to cell subsets within the T cell and B cell compartments, along with the frequency of monocytes (Fig. 4B and gating strategy shown in Fig. S2). From this highly comprehensive approach covering more than 110 distinct immunophenotyping measurements, we identified 29 subpopulations that differed in frequency (% of live) and 17 that differed in the mean signal intensity (MSI) of phenotypic markers (Fig. S3A, B). The frequency of total B cells was increased at W6 of HAMSAB supplementation and remained elevated after the 6-week washout (Fig. 4C and Fig. S3A). The increased frequency of B cells at W6 was mainly the result of an increase in naïve B cells identified as IgD^+^CD27^-^ (Fig. 4C and Fig. S3A). After the 6-week washout period, CD86 expression was downregulated on B cells gated as CD27^+^IgD^+^ IgM^hi^ (Fig. 4D and Fig. S3B). This CD27^+^IgD^+^IgM^hi^ subpopulation of human B cells has been described as phenotypically related to marginal zone B (MZB) cells [29, 30]. Downregulated CD86 expression on MZB cells was also observed in the spleen and pancreatic lymph nodes of HAMSA-fed NOD mice in which reduction of hyperactive antigen-presenting MZB cells correlated with protection against diabetes [5, 31]. After termination of the HAMSAB supplementation, we observed an increase in the frequency of atypical IgD^-^IgM^-^ B cells (Fig. 4E), which have been detected in the blood of patients with systemic lupus erythematosus (SLE) and have defective gut-associated MZB development [32].

### Increased immunoregulatory changes within T cells following HAMSAB supplementation

The total frequency of CD3^+^ T cells increased after HAMSAB supplementation, reaching a peak at W12 (Fig. 4E and Fig. S3A). This was reflected by an increase in effector memory (EM) and terminal effector memory CD45RA^+^ (TEMRA) cells within both CD4^+^ and CD8^+^ T cell compartments (Figs. 3A, 4A-D). EM and TEMRA were increased in both CD4^+^ regulatory T cells (Tregs) and non-regulatory conventional CD4^+^ T cells (Tconv) (Figs. S3A, S4B). In contrast, the frequencies of naïve and central memory (CM) Tconv, Tregs and CD8^+^ T cells were significantly reduced at W12 compared with baseline (Figs. S3A, S4B-D). At W12, we observed a higher expression of CD152 (cytotoxic T lymphocyte–associated protein 4 (CTLA4)) across EM and naïve Tconv and CD8^+^ T cell subsets (Figs. S3B and S4E). The fact that we found increased expression of inhibitory CTLA4 on Tconv and CD8^+^ T cells that expressed granzyme B and perforin (Grzb^+^Perf^+^) (Fig. 4F and Fig. S3B) suggests that HAMSAB may reprogram cytotoxic T cells. CTLA4 inhibits CD28-mediated T cell co-stimulation by binding and downregulating CD80/86 on antigen-presenting cells [33], which is consistent with the lower CD86 expression on MZB cells, plasmacytoid dendritic cells (pDCs) and myeloid/conventional dendritic cells (mDCs) at W6 and W12 (Figs. S3B and S4F). The frequency of monocytes was decreased at W12 follow-up (Figs. S3A, S3B and S4G). We found that HAMSAB supplementation was followed by increased expression of the coinhibitory molecule TIGIT (T cell immunoreceptor with Ig and ITIM domains), which in Tregs has been shown to suppresses pro-inflammatory Th1 and Th17 responses [34] (Fig. 4G). TEMRA Foxp3^+^ Tregs and CM Tconv CD4^+^ T cells expressing TIGIT were found to be increased at W6, while CD45RO^+^ Tconv cells expressing TIGIT were increased at both W6 and W12 (Fig. 4G). Remarkably, our results show that the immune system in adults with long-standing T1D was modulated towards immune regulation following HAMSAB supplementation, which persisted after the treatment stopped.

### Circulating pro-inflammatory markers are decreased, and oxidative phosphorylation is increased subsequent to HAMSAB supplementation

**Fig. 5:**
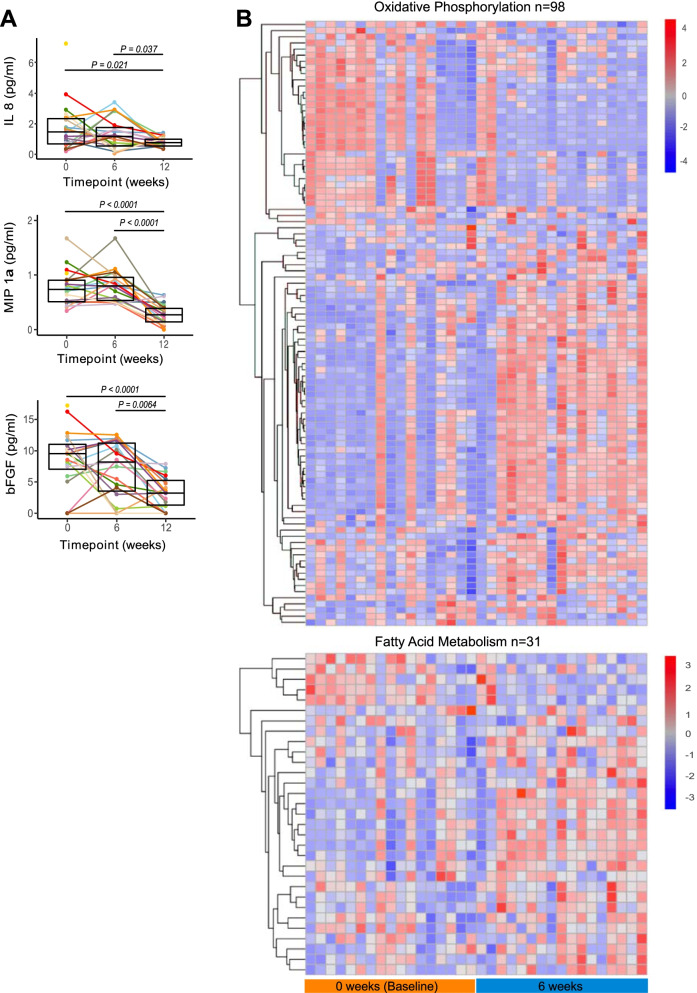
Circulating pro-inflammatory mediators are reduced at W12 in subjects following HAMSAB supplementation. **A** Serum IL-8, MIP1a, and bFGF concentrations detected by multiplex assay. Overall significance determined by GEE and pairwise differences between timepoints by estimated marginal means. Box plots show mean and upper and lower quartile ranges. **B** Hierarchical clustering of genes from fatty acid metabolism KEGG pathway gene set and from oxidative phosphorylation KEGG pathway gene set at baseline and 6 weeks of HAMSAB supplementation (*FDR* = 0.015 and 0.002, respectively). Columns represent individual subjects and rows represent individual genes in the pathway

We quantified the plasma concentrations of several pro-inflammatory and anti-inflammatory cytokines by multiplex bead array (Fig. 5A and Fig. S5). Plasma IL-8, MIP-1α, and bFGF were decreased following the 6-week washout period compared with baseline (Fig. 5A). IL-8 and MIP-1α are critical immune mediators associated with Th1 responses [35, 36]. The changes in immune cell types across the study suggested that the HAMSAB supplement might affect gene expression, given the potent metabolic epigenetic effects of SCFAs reported previously [5, 37, 38]. RNA-seq from whole blood comparing baseline and W6, together with gene set analysis, revealed that 52 KEGG pathways were upregulated (Table S3) including fatty acid metabolism (114 genes) and oxidative phosphorylation (31 genes), while no pathways were significantly downregulated at W6. Genes included in these pathways were not individually differentially expressed at FDR 0.05, consistent with our observation that only four genes overall showed a significant change at W6 (Table S4), but coordinated changes in pathways were evident (Fig. 5B, C and Table S3). Fatty acid metabolism has been suggested to play an important role in differentiation and regulation of T cell function [39]. Likewise, resting lymphocytes produce energy via oxidative phosphorylation and fatty acid oxidation, and rapidly shift to aerobic glycolysis in activated lymphocytes [40]. These changes are in line with the immunoregulatory phenotype observed in the patients during and after HAMSAB supplementation. Together, these data again indicate that HAMSAB supplementation is associated with changes in immune profiles towards an immunoregulatory phenotype.

### Evidence for interactions between microbiome, immune, and clinical parameters

**Fig. 6:**
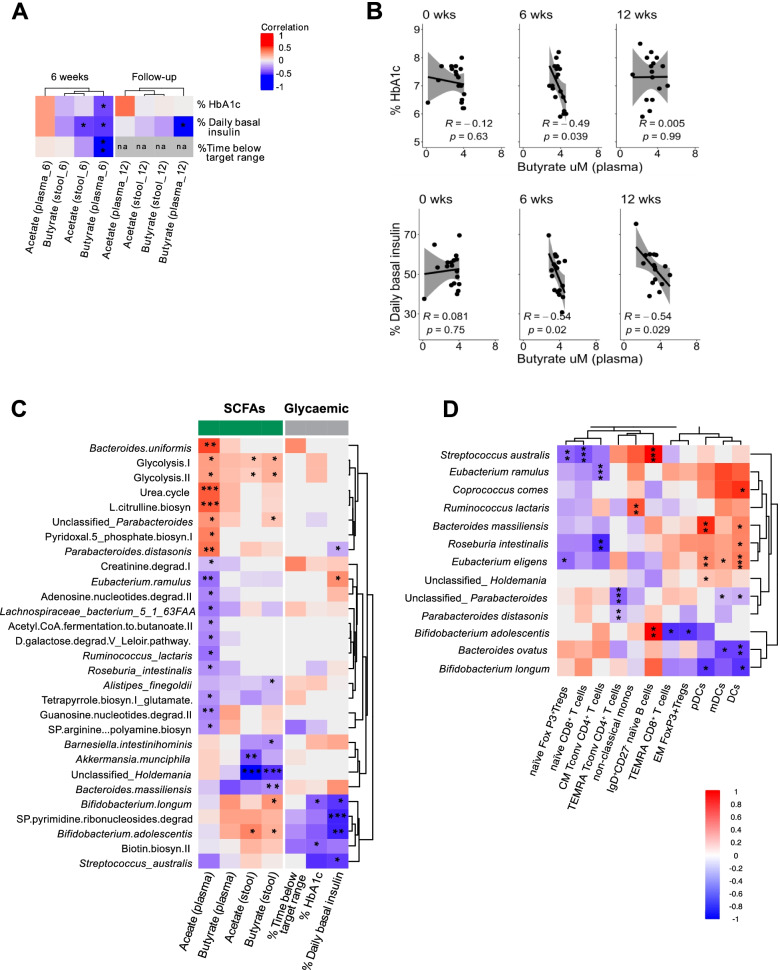
Increased SCFAs correlated with changes in glycemic control, commensal microbiota, and immune cell changes. **A** Heatmap of Pearson r values between relative abundance of SCFAs and clinical data. *Adjusted *P* < 0.05. **Adjusted *P* < 0.01. Stool and plasma short-chain fatty acids are hierarchically clustered based on Bray-Curtis dissimilarity. **B** Pearson r values at each timepoint between plasma butyrate, HbA1c, and daily basal insulin. Grey shading represents 95% confidence interval. **C** Heatmap of regression coefficients determined by GEEGLM between bacterial taxa and pathways with stool and plasma SCFAs and glycemic markers across all three timepoints. Bacterial pathways and taxa are hierarchically clustered based on Bray-Curtis dissimilarity. **D** Heatmap of significant regression coefficients across all three timepoints determined by GEEGLM between bacterial taxa that significantly changed across time (adj *P* < 0.05) and/or those correlated with SCFA and glycemic markers in (**C**) and significantly altered immune subsets (adj *P* < 0.05). *Adjusted *P* < 0.05, **adjusted *P* < 0.01, ***adjusted *P* < 0.001

Next, we investigated the relationship between SCFAs, clinical parameters, and metagenomic and immune phenotype data. At W6, plasma butyrate was negatively correlated with HbA1c, time below range, and basal insulin dose (Fig. 6A, B and Fig. S6A, S6B), the latter persisting beyond the 6-week washout period. Basal insulin dose and fecal acetate were also negatively correlated at W6 (Fig. S6A). There was no correlation between plasma acetate and glycemic variables (Fig. 6A) or between total daily insulin and SCFA concentrations at any timepoint (data not shown). Basal insulin dose negatively correlated with the relative abundance of *P. distasonis*, *B. longum, B. adolescentis*, and *Streptococcus australis* and with the nucleoside/nucleotide degradation pathway pyrimidine riobonucleoside degradation across timepoints (Fig. 6C and Fig. S6C). HbA1c negatively correlated with *B. longum* and biotin biosynthesis (Fig. 6C). These findings suggest that higher levels of *Bifidobacterium* taxa and biotin biosynthesis by the gut microbiota, supported by higher local acetate and butyrate availability, is associated with better glycemic control in adults with T1D.

The increases in SCFAs in stool and plasma were related to changes in gut microbiota composition (Fig. 6C). Stool butyrate was positively correlated with unclassified *Parabacteroides, B. longum* and *B. adolescentis,* while stool acetate positively correlated only with *B. adolescentis.* Stool butyrate was negatively correlated with *Alistipes finegoldii, Barnesiella intestinohominis,* unclassified *Holdemania* and *Bacteroides massiliensis,* and stool acetate negatively correlated with *Akkermansia munciphila* and unclassified *Holdemania* (Fig. 6C). Likewise, plasma acetate was positively correlated with taxa that were most strongly increased at week 6 (*B. uniformis,* unclassified *Parabacteroides*, and *P. distasonis*) and was negatively correlated with *E. ramulus*, *Lachnocpiraceae bacterium 5 1 63FAA*, *Ruminococcus lactaris*, and *Roseburia intestinalis* (Fig. 6C). Although plasma butyrate showed similar trends to stool butyrate, these were not significant after correction for multiple comparisons (Fig. 6C). Plasma acetate correlated positively with several bacterial pathways: amino acid biosynthesis pathways (urea cycle and L-citrulline biosynthesis) and a B6 vitamin biosynthesis pathway (Fig. 6C). Glycolysis I and II pathways were positively correlated with stool acetate and butyrate and plasma acetate. Plasma acetate was negatively correlated with amine/polyamine biosynthesis, nucleoside/nucleotide degradation, and fermentation and tetrapyrrole biosynthesis pathways (Fig. 6C). Hierarchical clustering of these correlations (Fig. 6C), clustered *B. longum*, *B. adolescentis*, *S. australis*, and biotin biosynthesis away from the taxa and pathways correlated with plasma acetate. This indicates that a different microbial community may be associated with those subjects in whom a high level of acetate entered the circulation compared with those whose plasma acetate remained low.

Changes in immune subsets particularly those involving B cells, TIGIT^+^RO^+^ Tconv cells, and non-classical monocytes were related to the increased concentrations of stool butyrate and plasma acetate (Fig. S6D). Further analysis of B cell subsets revealed that IgD^+^CD27^-^ naïve B cells positively correlated with *B. adolescentis* and *S. australis* (Fig. S6D). Naïve Tregs and naïve CD8^+^ T cells were negatively correlated with *S. australis.* Meanwhile, EM Tregs were negatively correlated with *B. adolescentis.* TEMRA Tconv cells were negatively correlated with *P. distasonis* and unclassified *Parabacteroides* (Fig. S6D). CM Tconv cells were negatively correlated with *E. ramulus* and *Roseburia intestinalis*. *E. eligens, Bacteroides massiliensis, Roseburia intestinalis*, and *Coprococcus comes* were positively correlated with total DCs, mDCs, and/or pDCs while *Bacteroides ovatus* and *B. longum* were negatively correlated with these subsets*.* In contrast, mDCs expressing CD86 were negatively correlated with *B. ovatus* (Fig. S6D and Fig. S4F)*.* Overall, these data suggested that increased SCFA production following HAMSAB supplementation is linked with immune–microbiome interactions.

## Discussion

This is the first human study investigating the effects of an acetate- and butyrate-modified starch supplement (HAMSAB) in adults with long-standing T1D. Subjects taking the HAMSAB supplement increased acetate, propionate, and butyrate in both stool and plasma, indicating metabolism of the starch by the gut bacteria. Changes in the gut bacterial composition and function included a reduction in carbohydrate degradation, promotion of gluconeogenesis, amino acid, and vitamin B2, B6, B7, and B9 biosynthesis pathways, consistent with SCFA utilization by the microbiota [28]. Remarkably, increased SCFAs following the HAMSAB supplement resulted in modulation of T cells, B cells, DCs, and monocytes towards a more regulatory immune phenotype. A correlation network associated stool acetate and butyrate, plasma butyrate, higher *Bifidobacterium* and biotin biosynthesis, lower HbA1c, and lower daily basal insulin requirements.

Subjects in our study consumed the HAMSAB supplement for 6 weeks, during which time they experienced no overall impacts on their diabetes management demonstrating the supplement was safe. While our study was not designed to determine efficacy, the observed correlations between increased SCFA and improved glycemic control were similar to what was found in human studies testing the effect of HAMS, type 4–resistant starches or high fiber from vegetables in subjects with type 2 diabetes or metabolic disease, as well as in healthy individuals [4, 17, 41]. Provision of a high-fiber diet to adults with long-standing T1D for 6 months resulted in decreased daily blood glucose and decreased hypoglycemic events compared with a low-fiber diet [42]. An increased high-fiber diet in the form of oligofructose-enriched inulin, administered to children with long-standing T1D for 12 weeks, did not change HbA1c or inflammatory markers, but the inulin-treated children had preserved C-peptide and increased relative abundance of *Bifidobacterium* compared with the placebo group [43]. While our study could not assess whether the effects observed were due to the additional acetate and butyrate, preclinical mouse models in T1D, infection, and cancer have shown the beneficial effects of added acetate or butyrate delivery compared with the starch (HAMS) alone [5, 10, 15]. Other studies have investigated the direct effect of oral postbiotics such as sodium butyrate changing the immune status in long-standing T1D and obese individuals compared with a placebo group [44, 45]. As we observed reduced inflammatory markers and marked immune changes following supplementation, this suggests long-term HAMSAB dietary supplementation via the gut microbiota may elicit further beneficial effects from increased SCFAs over and above those from high-fiber or the starch carrier itself or from delivery of soluble SCFA alone. Further studies comparing with a placebo group (HAMS supplementation) will allow assessment of whether HAMSAB is superior or has distinct effects on metabolic and immune outcomes.

HAMSAB supplementation was associated with significantly increased concentrations of SCFAs in stools and plasma. SCFAs are used as a source of energy by the host and the gut microbiota [46]. In humans, more than 95% of the bacterially produced SCFAs are absorbed by the colonocytes where butyrate is the major energy source [47–49]. Acetate is used in the synthesis of cholesterol, long-chain fatty acids, and amino acids such as glutamine and glutamate, and it is highly abundant in circulation. Meanwhile, propionate acts as a precursor of glucogenesis [50–52]. Propionate and butyrate are mostly cleared in the liver before reaching portal circulation, which explains their lower concentrations in peripheral blood compared with stools. Released SCFAs that pass through the liver also are metabolized in many other organs such as the brain, heart, kidney, muscle, and adipose tissue where they intersect with their immune system and metabolism [1, 53]. Thus, the detection of SCFAs in peripheral blood represents a good measure of their release by the gut microbiota with systemic functions, as shown in other human studies [4, 54, 55].

Increasing concentrations of acetate and butyrate after HAMSAB feeding was associated with protection against diabetes in NOD mice [5]. In our preclinical study, HAMSA diet led to a change in the overall composition of B cells, driven by a decrease in MZB cells with high CD86 and MHC-I expression. Meanwhile, the HAMSB diet drove an increase in regulatory T cell frequency and IL-10 production, and both diets reduced the proliferation of islet-specific T cells. Remarkably, these findings were similar to what we observed here, where MZB-like cells expressing CD86 decreased over time, and T cells expressing the inhibitory molecule CTLA4 increased. It has been shown that MZB cells with a regulatory phenotype are induced by the gut microbiota to restrain chronic inflammation and protect against arthritis [56]. Moreover, acetate has been shown to induce the immunosuppressive activity of regulatory B cells associated with reduced arthritis severity [57]. Hence, modulation of B cells towards a more regulatory phenotype observed in the patients at W6 of the HAMSAB delivery may have the potential to preserve pancreatic beta cells if trialed in recent-onset or preclinical disease.

T1D is a T cell–driven autoimmune disease and modulation of effector T cells is considered critically important for a successful therapy to halt the autoimmune attack. While we did not investigate islet-specific T cells, as individuals with long-standing T1D have a low frequency of such cells [58, 59], we observed an increase in T cells expressing the immune-regulatory molecule TIGIT. TIGIT is characteristic of exhausted or dysfunctional T cells and was also increased in the successful anti-CD3 and alefacept trials in T1D [60, 61]. Our results showing increased Treg-expressing CTLA-4 cells at W12 and reduced CD86 expression by antigen-presenting cells are consistent with previous reports that Treg-mediated CTLA4-dependent downregulation of CD80/CD86 on DCs could inhibit the rapid proliferation of pathogenic CD4^+^ T cells [62]. Of note, the most significant changes in immune profile occurred at 12 weeks, when the levels of SCFAs in plasma and stools remained significantly elevated compared with the baseline, which may suggest delayed yet sustained effects of the supplement on immune system regulation. Further studies using a longer period of dietary intervention may be needed to assess optimal effects and benefits of the HAMSAB supplement.

Analysis of the fecal microbiome demonstrated a substantial shift in both the composition, diversity, and function of the microbial community after the HAMSAB intervention. Other studies using specific resistant starches or dietary fiber have also shown decreases in the number of bacterial taxa due to differences in ability to metabolize resistant starch sources [24, 43, 63]. Major changes were specific to *P. distasonis,* which is a saccharolytic anaerobe involved in digestion of polysaccharides [64]. Two previous clinical trials that delivered HAMSB to healthy volunteers also observed increased abundance of *P. distasonis*, suggesting this is a common response to HAMS-modified starches [22, 23]. *P. distasonis* can produce acetate (though not a major producer) succinate and secondary bile acids [64, 65]. The health benefits of *P. distasonis* are variable and depend on the disease context. *P. distasonis* decreased weight gain, hyperglycemia, and liver disease in a high-fat diet mouse model [65]. In a colitis model, *P. distasonis* components reduced disease severity [66]. In human disease, *Parabacteroides* genera have variously been found to be increased in children at the onset of T1D [67] and in ankylosing spondylitis [68]. As such, it is not yet clear whether an increase in *P. distasonis* abundance following HAMSAB treatment will have an overall beneficial impact in the context of T1D, and further studies are needed to unravel the precise impact of this species, potentially using animal models.

The HAMSAB supplement promoted changes in *B. adolescentis*, which trended to increase while on the supplement, and was positively correlated with both acetate and butyrate concentration in the stool. Bifidobacteria do not produce butyrate themselves but are known for their mutualistic ‘cross-feeding’ behavior, whereby they produce lactate and acetate that support the presence of other butyrate-producing taxa [69]. Functional changes in the microbiota at 6 weeks included the ‘*Bifidobacterium* shunt’, which is linked to this cross-feeding pathway. We observed that stool concentrations of acetate, butyrate, and propionate all remained elevated at the 12-week follow-up timepoint. This suggests that a persistent effect on SCFA availability may remain after the intervention stopped. Analysis of food diaries and macronutrient intake suggested this was not due to any altered dietary patterns over the course of the study. Rather, it is likely that either SCFA production or utilization patterns changed. As we did not find any significant differences in the composition of the microbiota or the pathways present in the genomes of the bacteria at 12 weeks compared with baseline, this may represent a functional reprogramming of the taxa present or an alteration in the host consumption of SCFAs. A limitation of functional inference from metagenomic sequencing data is that it does not determine which pathways are actually active in the bacteria at any given time. Of note, the microbial pathway linked to fermentation to butanoate significantly increased at 12 weeks compared to 6 weeks, indicating a switch to SCFA production rather than utilization at 12 weeks. More direct measures of the host and bacterial activity are needed to clarify the mechanism, and such studies are ongoing.

The major microbial functional pathways that were altered at the 6-week timepoint included increased glycolysis/decreased carbohydrate degradation, increased amino acid synthesis and increased B group vitamin and cofactor biosynthesis. The changes in carbohydrate metabolism suggest that the SCFAs released from HAMSAB were used as an energy source by the bacteria. *In vitro*, bacteria grown on acetate rather than glucose upregulate the TCA cycle and gluconeogenesis, consistent with our findings [10, 28]. The TCA cycle generates the precursor molecules that are required for the biosynthesis of nucleotides, amino acids, cofactors, and vitamins with beneficial properties for health. The biotin (vitamin B7) biosynthesis II pathway, which increased at week 6, clustered with *Bifidobacterium* and negatively correlated with HbA1c. *Bifidobacterium* does not possess biotin synthesis ability themselves; however; their establishment in the gut environment (particularly *B. longum)* has been suggested to augment the production of the biotin precursor pymelate, which is metabolized into biotin by other species such as *Bacteroides uniformis* [70–72]. Biotin is an NFκB inhibitor with anti-inflammatory effects on the immune system, suppressing production of inflammatory cytokines [73].

Interestingly, features of the microbiota that correlated with improved glycemic control were linked to stool SCFAs and plasma butyrate concentrations but not with plasma acetate. However, only a subset of subjects increased acetate levels systemically, whereas almost all increased acetate in their stool. We speculate that this may be related to a difference in the host’s ability to absorb acetate, perhaps due to differences in intestinal permeability or differential utilization of acetate. Individuals with T1D have increased intestinal permeability compared with healthy subjects, although this characteristic is highly variable [74–76]. Furthermore, the microbiota associated with T1D are also highly variable, which may result in a different response to HAMSAB. A longer period of HAMSAB dietary intervention would help resolve these individual differences and potentially achieve greater effects of the HAMSAB supplement.

## Conclusion

The study was designed to determine the safety of the supplement in the context of T1D and it did not include a placebo control group. Although the explorative outcomes provided significant insights into the effects of HAMSAB supplementation on microbiota composition and immune status, the changes observed are associative from comparisons within participants over time. In addition, the intervention used was only 6 weeks, which is a relatively short timeframe to look at changes in markers such as HbA1c (usually looked at over 3 monthly intervals) or C-peptide (usually assessed over 12-month intervals). A follow-up placebo-controlled trial powered to investigate efficacy will be needed to determine casual effects from the HAMSAB supplement in a larger study and longer period of intervention to better determine any impact on glycemic control.

We have shown that HAMSAB supplementation remodels the gut microbiota and significantly impacts the immune system. Moreover, our study has validated our preclinical results in the NOD mouse, indicating that the HAMSAB supplement is a physiological and efficient way to modulate microbiota–host interactions in T1D. This trial provides the necessary data to allow for an appropriately powered, double-blinded, placebo-controlled randomized controlled trial in T1D. If efficacious, an anti-inflammatory dietary HAMSAB intervention should be a widely acceptable, noninvasive and highly accessible means of preserving beta cell function in individuals with newly diagnosed T1D or in those at high risk of the disease.

## Methods

### Participants and study design

The trial was a single-arm pilot trial of the HAMSAB supplement administered twice daily as part of the usual diet for 6 weeks with follow-up at 12 weeks in adults with T1D. Inclusion criteria included 18 to 45 years, males and females; clinical diagnosis of T1D for at least 6 months; HbA1c ≤ 8.5%, and stable disease management. Exclusion criteria included pregnancy or lactation, use of diabetes medications other than insulin, hypoglycemia unawareness, concomitant disease or treatment that may impact on glycemic control, insulin requirements or other outcome measures, history or symptoms of gastrointestinal disease or malabsorption, any known conditions that could be associated with difficulty complying with the trial protocol, weight below 50 kg or above 120 kg, liver or renal disease, use of sensor-augmented insulin therapy, unwillingness to maintain their normal stable diet during the study and/or following a restrictive diet that would impact their ability to take the supplement, and recent (previous 6 weeks) or anticipated antibiotic use during the study.

Participants attended 4 visits at the Charles Perkins Centre Clinical Research Centre (W0, W3, W6, and W12). In the week prior to each study visit, participants wore a blinded continuous glucose monitor (Abbott Freestyle Libre Pro) for 14 days (up to W6). Optimal continual glucose monitoring (CGM) target in between 3.9-10 mmol/L. Participants completed: a 3-day food diary (2 weekdays and 1 weekend day via paper or smartphone app [Easy Diet Diary Research, Xyris], baseline and W6 only), a 7-day insulin logbook only if using multiple daily injections, otherwise insulin dosing history recorded from insulin pump, and the HAMSAB supplement dosing logbook (up to W6). Compliance with intervention was evaluated by self-reported log-books and measuring the returned unused supplement. Participants collected and froze a stool sample 24 h prior to their appointment and completed three online questionnaires: a validated 18-item, 5-point Likert scale Gastrointestinal Quality of Life Index Questionnaire [77] (gastrointestinal symptoms (i.e., bloating, constipation, and flatulence) measured as score range: 0–64, with higher scores indicating less gastrointestinal symptoms), a 10-item Diabetes-Specific Symptom Questionnaire (score range: 0–36 with higher scores indicating higher QoL), and an 8-item HAMSAB supplement feedback questionnaire to assess its palatability and acceptability. The HAMSAB supplement is a high-amylose corn starch acetylated and butyrylated (Ingredion Incorporated, Bridgewater, NJ, USA). To improve tolerability, participants commenced with 10 g/day and increased the dose in 10-g increments every 48 h until 40 g per day was reached. If the increased dose was not tolerated, the participant was to remain on the previous dose for an additional 48 h before attempting the increased dose again. If that dose was not tolerated after the third attempt (or on the judgement of the investigators), they were to remain on the previous dose for the remainder of the intervention. Participants were asked to return any unused HAMSAB supplement at the next visit.

### Laboratory measures

Blood and urine samples collected from participants at W0, W3, and W6 (*n* = 20, 19, and 19, respectively) were analyzed for serum urea, creatinine, electrolytes, liver function tests, bicarbonate, triglycerides, and urinary albumin. Urinary pH analyzed by dipstick on-site by trial coordinator at time of collection. Food diaries were analyzed using nutrient analysis software (FoodWorks Professional 9, Xyris) to assess changes in food/nutrient intake. Continuous glucose monitoring data was included for all days where the monitoring was worn for a full 24-h period (i.e., no partial days included). A Mixed Meal Tolerance Test (MMTT) was conducted at baseline, 6 and 12 weeks. Participants fasted for 8–12 h and no bolus insulin dosed with the test drink or in the 3 h prior. Usual basal insulin was allowed. The test was only performed if the fasting blood glucose was between 4 and 11 mmol/L as described previously [78]. The test meal was a standard mixture of fat, carbohydrate, and protein (Ensure; Nestle Health Care Nutrition, Inc). Venous blood samples were collected at 0, 30, 60, 90, and 120 min. Stimulated C-peptide was measured by ELISA (Sigma-Aldrich, RAB1389).

### SCFA analysis

Plasma samples were collected at baseline, W3, W6, and W12 (*n* = 20, 19, 19, and 18, respectively) and stool samples at baseline, W6, and W12 (*n* = 20, 19, and 18, respectively) and stored at – 80 °C until processing. SCFA acetate, propionate, and butyrate were measured in duplicates by gas chromatography after liquid–liquid extraction. Plasma SCFAs were directly measured by specialized polar phase gas chromatography–mass spectrometry (GC-MS) Phenomenex Zebron ZBFFAP column (Phenomenex, Torrance, CA, USA). Plasma (200 μL) was mixed with internal standards (50 μL of 200 μM heptanoic acid internal standard and 50 μL of 10% sulfosalycilic acid). Samples were mixed with 30 μL of 0.2 M NaOH, centrifuged, and let dry at room temperature. The residue was redissolved in 30 μL of 1 M phosphoric acid. Stool samples were mixed with internal standards solution and centrifuged. One hundred microliters of supernatant was filtered and analyzed on an Agilent 7890A gas chromatograph (Agilent Technologies, Santa Clara, CA, USA). Peaks were detected with a flame ionization detector at 210 °C and identified against calibration standards over the range 0 to 400 mM.

### Mass cytometry

PBMC were isolated from blood collected at baseline, W6, and W12 (*n* = 20, 19, and 18, respectively) by Ficoll-paque (GE Healthcare, Uppsala) centrifugation, cryopreserved in 10% dimethyl sulfoxide, 20% fetal calf serum (FCS) and RMPI-1640 (Thermo Fisher Scientific, Waltham) and stored in vapor-phase liquid nitrogen. All samples collected were thawed in batches, washed with RMPI-1640 (Thermo Fisher Scientific, Waltham), supplemented with 10% heat inactivated FCS and benzonase, and subjected to immunophenotyping analysis by mass cytometry. A panel of 27 metal-tagged monoclonal antibodies supplied by the Ramaciotti Facility for Human Systems Biology was used for analysis (Data Table S5). The gating strategy is shown in Data Fig. S2. Unlabelled antibodies were purchased in a carrier protein–free format and conjugated with the indicated metal isotope using the MaxPAR antibody conjugation kit (Fluidigm). For live–dead cell distinction, PBMCs were stained with 1.25 μM cisplatin. Cells were incubated for 30 min initially with anti-CD45 antibodies conjugated to various metals, which facilitated barcoding and pooling of samples from multiple timepoints for each participant together prior to staining with the remaining antibodies targeting surface antigens. Cells were then fixed and permeabilized with FoxP3 staining kit (eBiosciences, San Diego) and stained with FoxP3 antibody. Cells were fixed in 4% paraformaldehyde containing DNA intercalator (0.125 μM iridium-191/193; Fluidigm). After multiple washes, cells were diluted in MilliQ water containing 1:10 diluted EQ beads (Fluidigm) and acquired at a rate of 200–400 cells/s using a CyTOF 2 Helios upgraded mass cytometer (Fluidigm). All Helios data were normalized using the processing function within the CyTOF acquisition software based on the concurrently run EQ four element beads. Data analysis was performed using FlowJo version 10.4 software (FlowJo, LLC, Ashland, OR, USA). Samples were pre-gated on DNA^+^, live, CD45^+^ cells, and exported for further analysis.

### Metagenomic sequencing and data processing

Stool samples from participants were collected at baseline, W6, and W12 (*n* = 20, 19, and 18, respectively), and DNA was extracted from 0.2 g stool using a repeated bead beating method [79]. Briefly, bacteria were lysed and homogenized by bead beating (0.5 mm zirconia beads) with lysis buffer containing 4% SDS, 500 nM NaCl, 60 mM Tris-HCL, and 50 mM EDTA using a Precellys24 homogenizer (Bertin Technologies) for 3 × 60 s cycles at 5000 rpm with 15-s rests. Bacterial lysis was enhanced by further incubating at 70 °C for 15 min. RNA contamination was removed by incubation with RNase, and protein was digested with proteinase K. DNA was then extracted on a Promega Maxwell DX 16 for automated nucleic acid extraction using a Maxwell 16 LEV Blood DNA kit (Promega). Shotgun metagenomics library preparation and sequencing was performed by Genewiz using a NovaSeq platform (Illumina) obtaining sequence depth between 12.9 and 20 gb (43,000,000–69000000 reads per sample). Low-quality reads and adaptors were removed using Trimmomatic v 0.69 [80]. Kneaddata v0.73 (http://huttenhower.sph.harvard.edu/kneaddata) human and bacterial 16S sequence contaminants were removed and paired-end reads aligned. Bacterial taxonomic and functional identifications were determined using MetaPhlAn 2.0 [81] and HUMAnN2 [82], respectively. Bacterial taxa and functions that were not present in more than 40% of samples from one timepoint and with a total relative abundance less than 0.01% were removed for statistical analysis. Bacterial relative abundances were then log2 transformed. Two samples from one subject at baseline and W6 were not included in the metagenomic analysis as DNA failed to meet quality requirements.

### Multiplex assay

Plasma samples were isolated from blood collected at baseline, W6, and W12 (*n* = 20, 19, and 18, respectively), and inflammatory markers were analyzed by multiplex immunoassay using Bio-plex 200 system (Bio-rad, CA, USA) as per the manufacturer’s instructions. Circulating levels of cytokines were measured in duplicates by Bio-Plex Pro Human Cytokine 27-plex Assay (Catalog # M500KCAF0Y) including interleukin (IL)-1β, IL-1ra, IL-4, IL-7, IL-8 (chemokine CXCL8), macrophage inflammatory protein (MIP)-1α (CCL3), MIP-1β (CCL4), macrophage chemoattractant protein (MCP)-1 (CCL2), basic fibroblast growth factor (bFGF), interferon (IFN)-γ inducible protein (IP)-10 (CXCL10), IL-13, IL-9, and tumor necrosis factor (TNF)-α. C-peptide was measured using a Bio-Plex Pro^TM^ RBM Human Metabolic Panel 1 (Catalog # 171AMR1CK).

### RNA seq

Whole blood samples from participants were collected at baseline and W6 (*n* = 20 and 19, respectively) directly into Paxgene or Tempus tubes containing RNA stabilizers then stored at – 80 °C. RNA was purified according to the manufacturers’ instructions. Briefly, crude RNA was pelleted by centrifuging at 5000*g* for 15 min at 4 °C. The pellet was washed, resuspended, and treated with protease and DNase to remove contaminants. Finally, magnetic RNA-binding beads were added to capture RNA followed by additional washing and RNA elution. The purified RNA was checked for quality using Tapestation 4200 platform. Samples from two patients were not included in RNA-seq experiment due to the poor RNA integrity at either baseline or W6. Next-gene sequencing was performed by Genewiz using Illumina NovaSeq platform obtaining sequence depth of 50 million reads/sample. Transcriptome analysis was performed in samples from W0 and W6 for paired analysis.

### General statistics and reproducibility

Graphs were generated using R package ggplot2. Overall statistical significance was determined using geepack with pairwise differences identified by the package emmeans. *P < 0.05* was considered statistically significant. Total *n* refers to the number of biologically independent samples in each group. Box plots show mean and upper and lower quartile ranges, and bar plots show mean and standard deviation. In more detail, changes in clinical parameters, safety, tolerability SCFAs, microbiota, immune phenotype, and inflammatory measures over time were examined using generalized estimating equations (GEE) linear regression modelling unless otherwise stated. The GEE approach handles repeated measures and unbalanced designs whereby all observations with completed data at least one timepoint are included in analyses. All GEE models used robust standard error estimation, and repeated measurements over time were handled with unstructured correlation matrix. The linear regression assumptions of normality and homoscedasticity were checked prior to analyses to assess the suitability of a linear model. Analyses were performed in SPSS Version 25 software (IBM Corp. 1989, 2017).

For microbiota analysis, alpha diversity was calculated using the Inverse Simpson diversity index (Vegan, R package). Multivariate sparse partial least squares discriminant analyses accounting for repeated measures, quantified beta diversity between timepoints. Significant differences in beta diversity was determined using PERMANOVA (Adonis, R package). Differences in alpha diversity, taxa relative abundance, and short-chain fatty acids (acetate, butyrate, and propionate) across timepoints were determined using generalized estimating equations (GEE), adjusting for multiple correction using the Benjamini-Hochberg approach (GeePack, R package). Estimated marginal means determined significant pairwise differences and includes a Tukey adjustment for multiple testing. Regressions across all timepoints between bacteria, SCFA’s and glycemic condition indices were performed using GEE generalized linearized modelling again adjusting for multiple correction using the Benjamini-Hochberg approach (GeePack, R package). Regression coefficient of zero indicates that the linear fit is worse than the null hypothesis of zero (or straight line). Correlations between glycemic condition and SCFA’s for each timepoint were performed using Pearson’s regression (R cran). Differences in percent live and mean signal intensity of immune populations across timepoints were determined using multivariate partial least squares discriminant analyses (PLS-DA), accounting for repeated measures. Significance was determined using PERMANOVA. The contribution of specific immune populations to the variation in immune markers between timepoints was determined by analyzing their contribution to the components in the PLS-DA and using univariate GEE analyses.

For RNA-seq, statistical analysis of the quantified gene expression was performed using the edgeR v3.28.1 [83] and RUVSeq v 1.0.0 [84] Bioconductor packages within R statistical software. Differential expression analysis was performed comparing all samples at baseline with all samples at W6 taking account of the paired nature of the data and the tube used for sample collection (Paxgene or Tempus). The RUVSeq package was used to remove unwanted variation, employing the RUVg function and using a set of 1000 of the least variable genes as control genes. Gene set enrichment analysis was performed using the camera function of limma v. 3.42.2 [85] to determine regulated pathways at W6.

## Supplementary information

**Additional file 1: Fig. S1.** sPLS-DA plot loadings indicating the contribution of each taxa. **Fig. S2.** Gating Strategy for identification of immune cell phenotypes by CyTOF. **Fig. S3**. Immune cell phenotype changes following HAMSAB treatment. **Fig. S4**. Immune cell phenotype changes following HAMSAB treatment. **Fig. S5**. Circulating pro-inflammatory and anti-inflammatory cytokines measured in subjects at baseline, W6 and W12 following HAMSAB supplementation. **Fig. S6.** Correlations between concentration of SCFAs, clinical parameters, relative abundance of bacterial communities and significant changes in immune cell subsets. **Table S1**. Nutritional and gastrointestinal changes across time. **Table S2**. Functional pathways encoded by the gut microbiota changed after taking dietary supplement. **Table S3**. Upregulated KEGG pathway gene sets identified using the camera function of limma following 6-weeks of HAMSAB supplementation. **Table S4**. Results of differential gene expression testing with EdgeR and RUVseq comparing baseline and week 6. **Table S5.** Mass cytometry panel used for immunophenotyping.

## Data Availability

Metagenomic data is deposited in the European Nucleotide Archive (ENA) (accession codes PRJEB45815, ERP129973). RNA-seq data is available in the GEO database (accession code GSE176230).
