# Supplementary material for: Metabolite-based dietary supplementation in human type 1 diabetes is associated with microbiota and immune modulation
Source: Microbiome. 2022 Jan 19;10:9. doi: 10.1186/s40168-021-01193-9 (PMC8772108; doi:10.1186/s40168-021-01193-9)
Supplement: Supplementary file 2 — Additional file 1: Fig. S1. sPLS-DA plot loadings indicating the contribution of each taxa. Fig. S2. Gating Strategy for identification of immune cell phenotypes by CyTOF. Fig. S3. Immune cell phenotype changes following HAMSAB treatment. Fig. S4. Immune cell phenotype changes following HAMSAB treatment. Fig. S5. Circulating pro-inflammatory and anti-inflammatory cytokines measured in subjects at baseline, W6 and W12 following HAMSAB supplementation. Fig. S6. Correlations between concentration of SCFAs, clinical parameters, relative abundance of bacterial communities and significant changes in immune cell subsets. Table S1. Nutritional and gastrointestinal changes across time. Table S2. Functional pathways encoded by the gut microbiota changed after taking dietary supplement. Table S3. Upregulated KEGG pathway gene sets identified using the camera function of limma following 6-weeks of HAMSAB supplementation. Table S4. Results of differential gene expression testing with EdgeR and RUVseq comparing baseline and week 6. Table S5. Mass cytometry panel used for immunophenotyping. [file 40168_2021_1193_MOESM1_ESM.pdf]

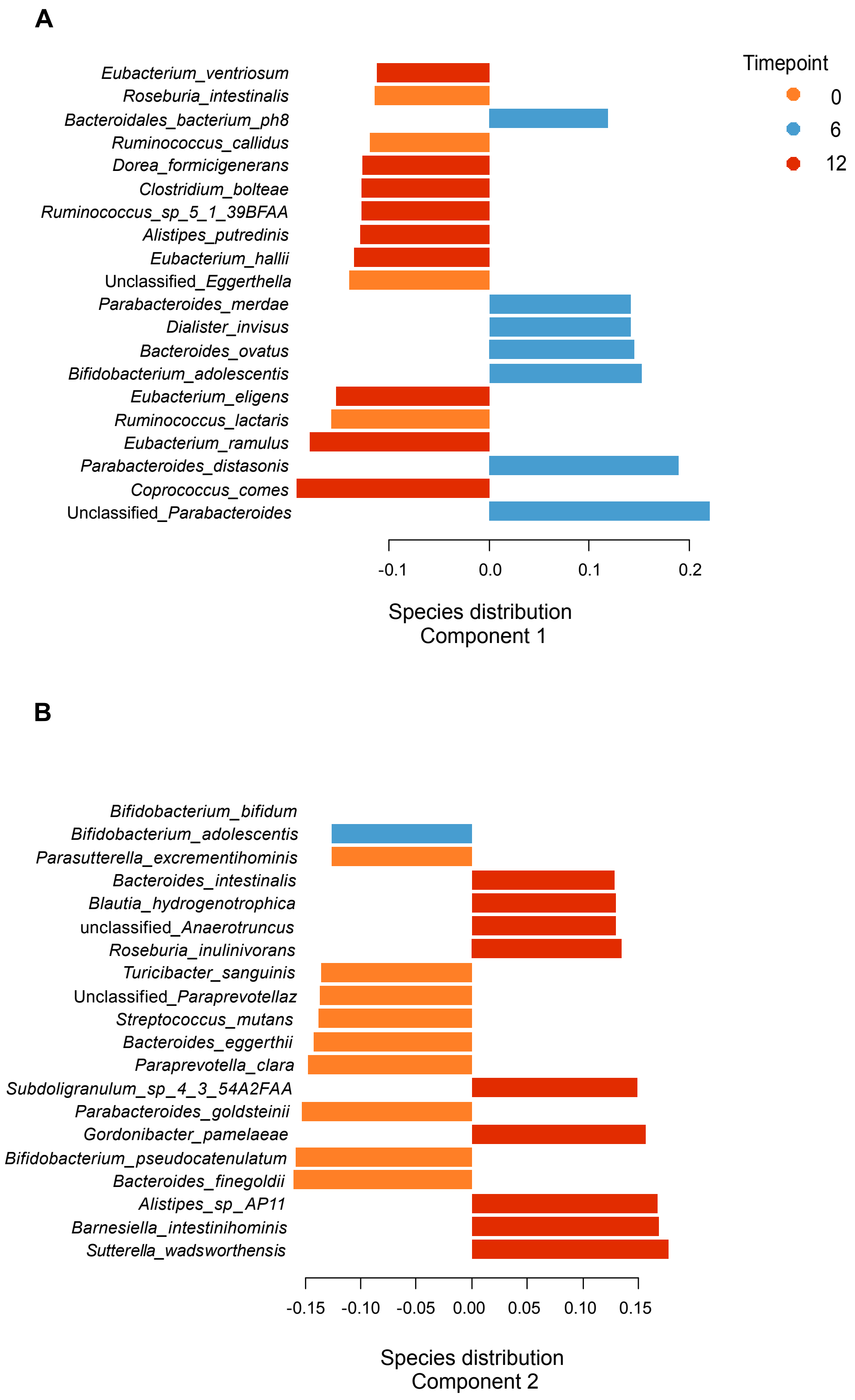

Fig. S1. sPLS-DA plot loadings indicating the contribution of each taxa. Color corresponds to the timepoint in which the taxa is most abundant. Loadings are associated with Fig. 3B (taxa).
(A) Component 1 species distribution
(B) Component 2 species distribution

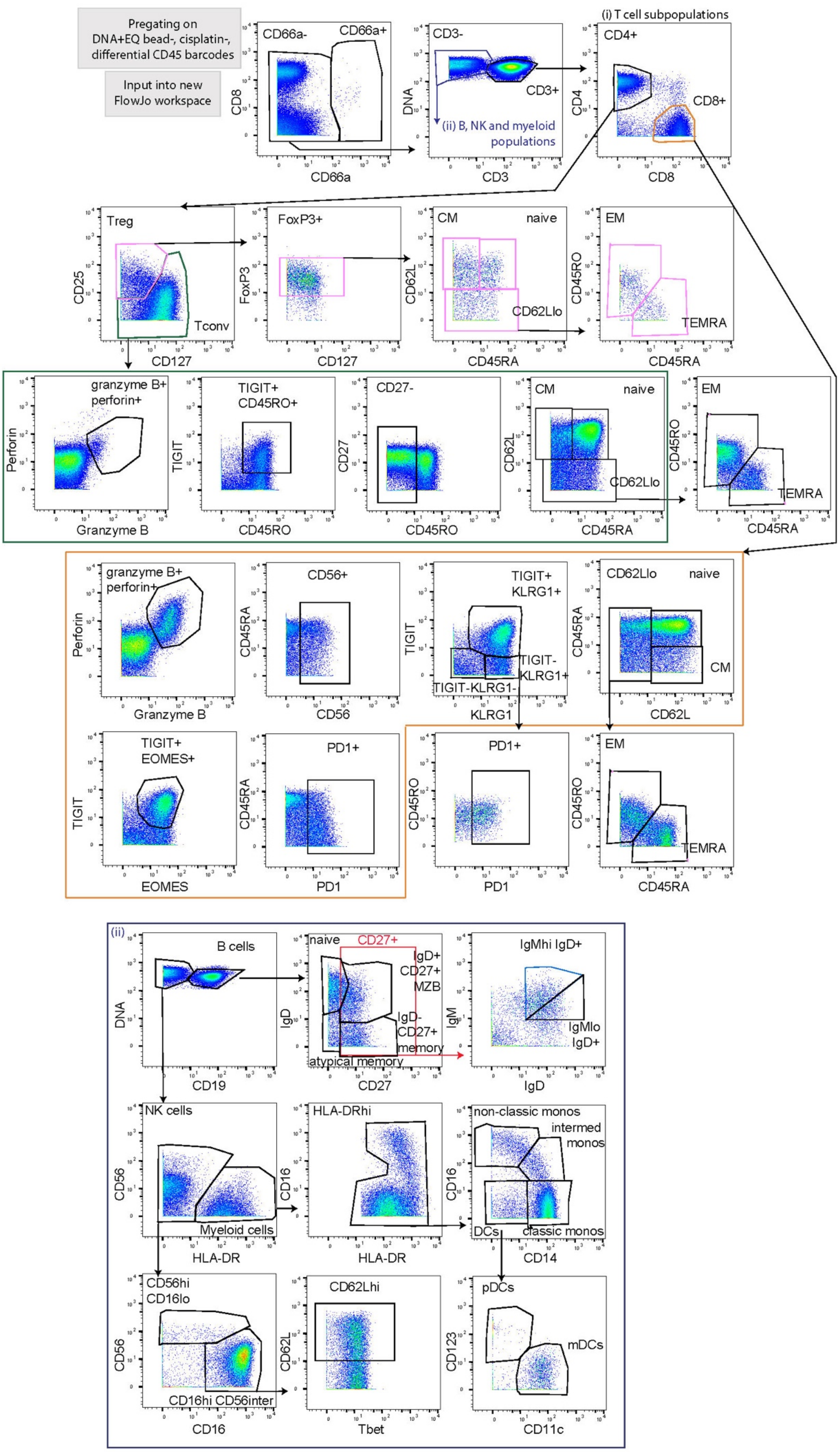

Fig. S2. Gating Strategy for identification of immune cell phenotypes by CyTOF.

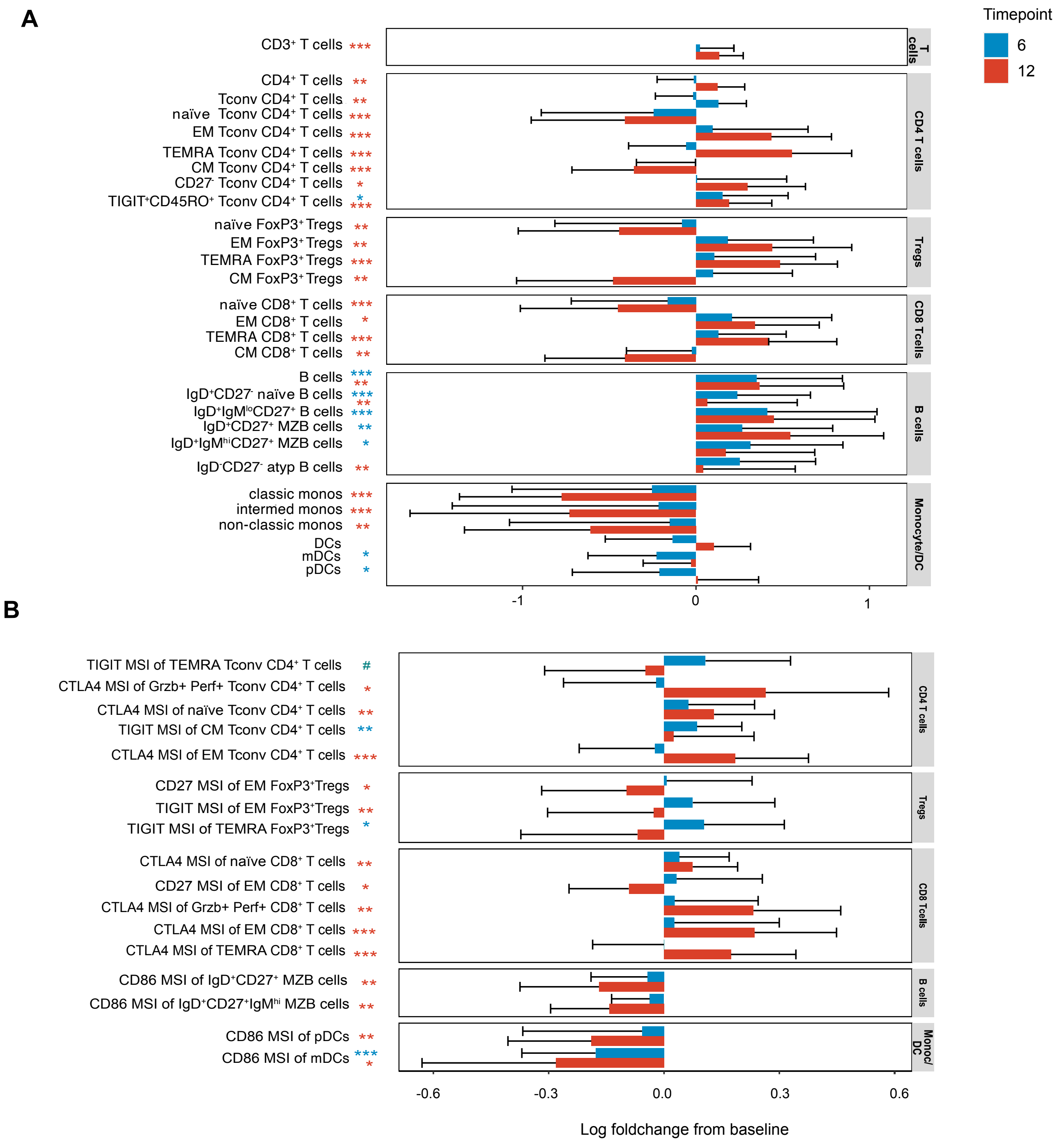

Fig. S3. Immune cell phenotype changes following HAMSAB treatment.
Log foldchange of frequency of live cells.
Log foldchange of MSI from baseline. Asterisk represents GEE significance of changes in % live and MSI across timepoints: blue asterisk W0 vs W6 and red asterisk W0 vs W12. Mean and standard deviation are shown. #adjusted $P < 0.1$, *adjusted $P = 0.1$- 0.05, **adjusted $P = 0.05$-0.001 ***adjusted $P < 0.001$.

Bell, Saad & Tillett, et al., Fig. S4

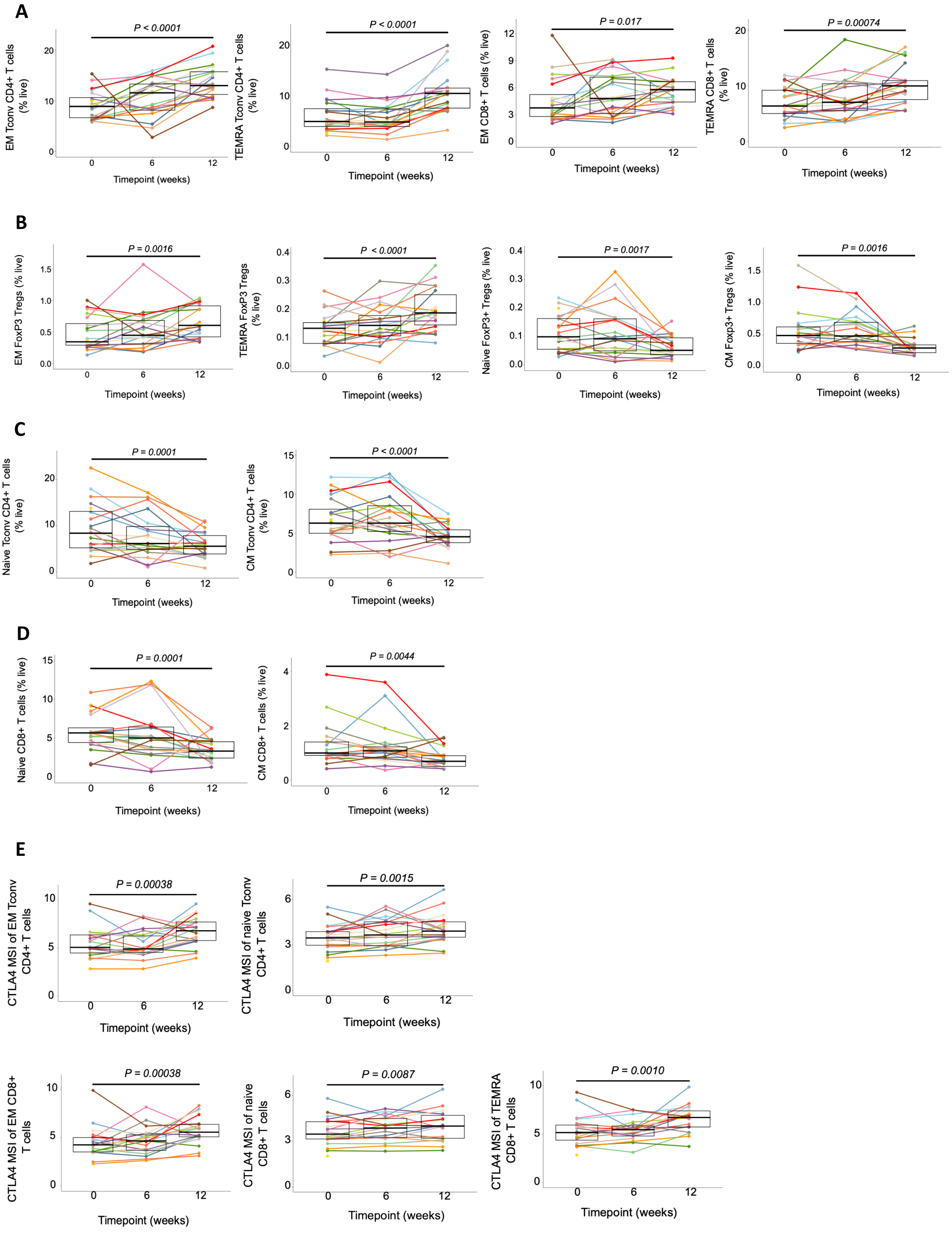

F

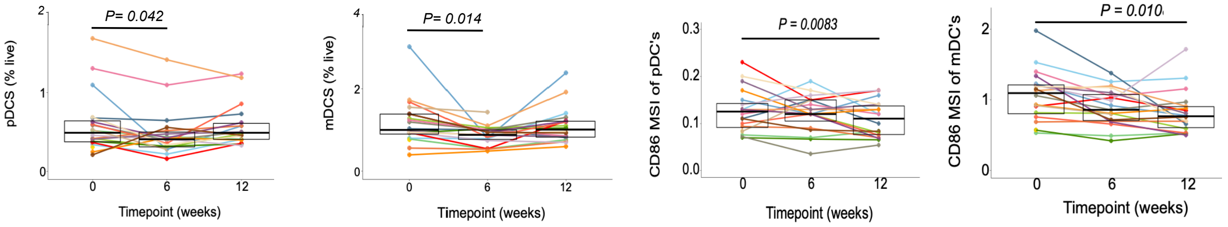

G

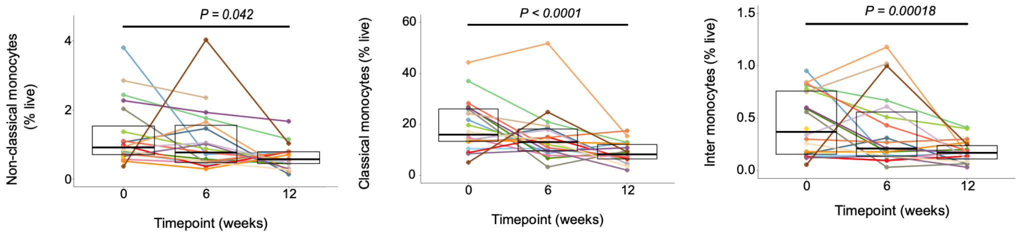

Fig. S4. Immune cell phenotype changes following HAMSAB treatment.
Mass cytometry of PBMC was used to determine total frequency (within live cells) or MSI of:
(A) EM Tconv cells, Tconv TEMRA cells, $CD8^{+}$ EM and $CD8^{+}$ TEMRA cells.
(B) EM Tregs, TEMRA Tregs, naïve Tregs, CM Tregs.
(C) naïve and CM Tconv cells.
(D) Naïve and CM $CD8^{+}$ T cells.
(E) EM and naïve Tconv expressing CTLA-4 and EM, naïve and TEMRA $CD8^{+}$ T cells expressing CTLA4.
(F) Frequency of total pDCs, mDCs and MSI of CD86.
(G) Frequency of total non-classical, classical and intermediate monocytes. Coloured dots represent each subject. Box plots show mean and upper and lower quartile ranges. Significance determined by GEE. Adjusted *P* values are (6W vs W0) or (12W vs W0). Gating strategy shown in Fig. S2.
.

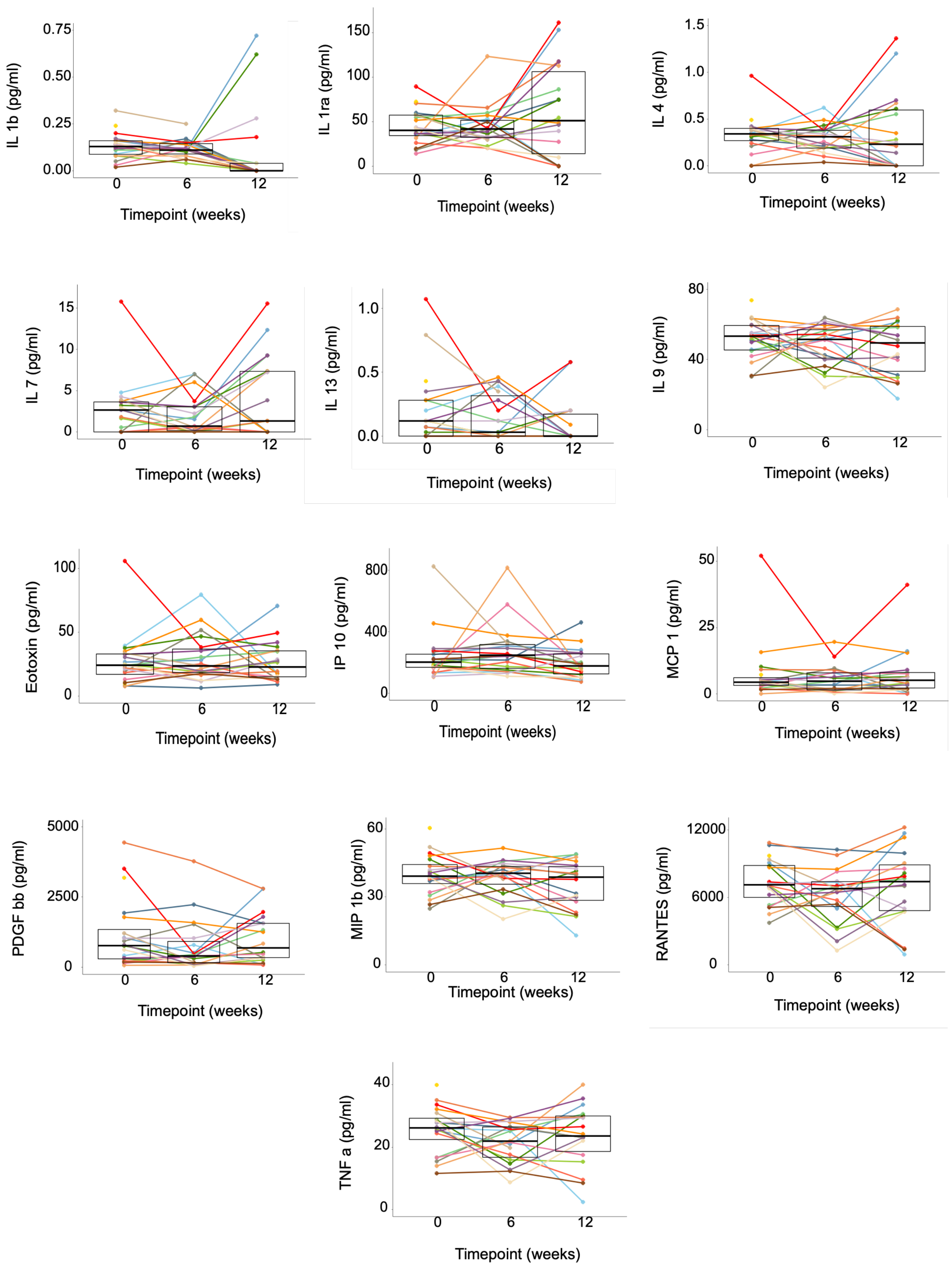

Fig. S5. Circulating pro-inflammatory and anti-inflammatory cytokines measured in subjects at baseline, W6 and W12 following HAMSAB supplementation. Overall significance determined by GEE. Adjusted *P* >0.05 (not significant in all the groups). Coloured dots represent each subject. Box plots show mean and upper and lower quartile ranges .

Bell, Saad & Tillett, et al., Fig. S6

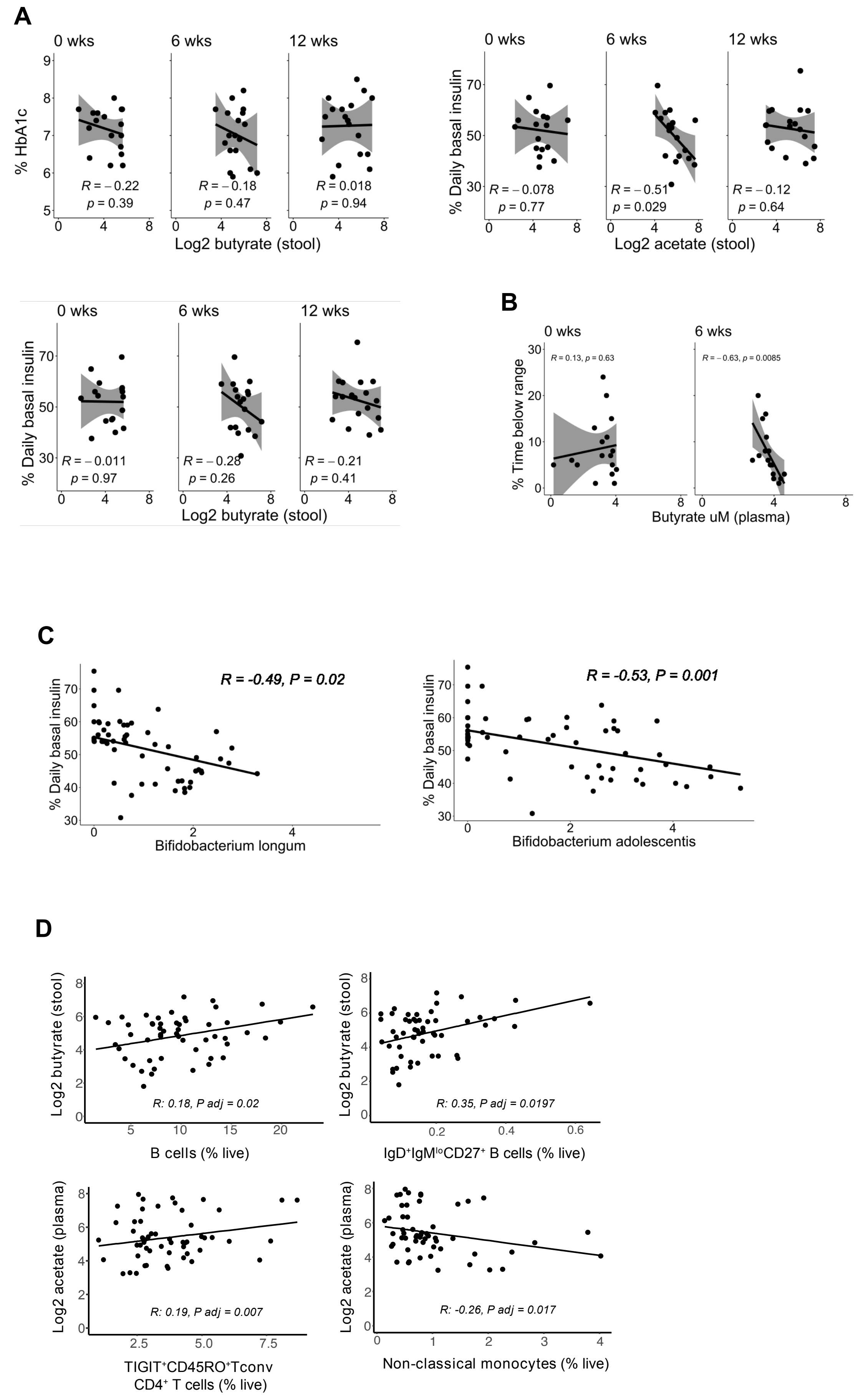

Fig. S6. Correlations between concentration of SCFAs, clinical parameters, relative abundance of bacterial communities and significant changes in immune cell subsets.

(A) Pearson r values at each timepoint between plasma butyrate and stool acetate and butyrate with daily basal insulin.

(B) Pearson r values at each timepoint between plasma butyrate and stool acetate and butyrate with time below target continual glucose monitoring range (70 – 180 mg/dL). Grey shading represents 95% confidence intervals.

(C) GEE generalized linear modelling regressions accounting for repeated measures between Log2 relative abundances of *Bifidobacterium longum* and *adolescentis* and daily basal insulin. (D) Regression modelling between frequency of total live immune cells with Log2 relative abundances of butyrate and acetate in plasma and stools using GEE generalised linearised modelling again adjusting for multiple correction using the Benjamini-Hochberg approach. Adjusted *P* values between the groups are shown on the graphs .

A. Gastrointestinal Symptoms and nutritional changes

Questionnaires	Visit 1 (baseline)	Phone call (week 1)	Visit 2 (week 3)	Visit 3 (week 6)	Visit 4 (week 12)
**Gastrointestinal Quality of Life Index (GIQLI), (P = 0.002)**	54 ± 6 (n=20)	57 ± 4 (n=20), P = 0.003	55 ± 4 (n=20)	54 ± 7 (n=20)	57 ± 3 (n=19), P = 0.003
**Diabetes-Specific Quality of Life**	27 ± 5 (n=20)	31 ± 3 (n=20)	29 ± 4 (n=20)	29 ± 5 (n=20)	28 ± 6 (n=19)

B. Macronutrient intake did not differ between timepoints

Nutrition Intake	Visit 1 (baseline)	Visit 3 (week 6)
**Energy (kJ [kCal])**	8746 ± 2033 [2092 ± 486] (n=20)	8686 ± 2469 [2078 ± 591] (n=18)
**Carbohydrate (% total energy intake)**	37.3 ± 9.3 (n=20)	37.0 ± 7.1 (n=18)
**Protein (% total energy intake)**	15.9 ± 4.0 (n=20)	16.7 ± 4.0 (n=18)
**Total Fat (% total energy intake)**	41.0 ± 38.5 (n=20)	38.5 ± 4.7 (n=18)
**Saturated Fat (% total energy intake)**	14.5 ± 2.4 (n=20)	14.3 ± 3.4 (n=18)
**Fibre* (g)**	24.4 ± 7.0 (n=20)	26.4 ± 10.3 (n=18)
**Glycemic Index (%)**	54.2 ± 5.1 (n=20)	
**Glycemic Load (g)**	108.6 ± 38.4 (n=20)	

Table S1. Nutritional and gastrointestinal changes across time.
(A) Gastrointestinal and diabetes-specific quality of life assessments. Overall significance determined by GEE, adjusted $P < 0.002$. $P < 0.003$ (visit week 1 and week 4 vs baseline).
(B) Macronutrient intake across the study. Not significant.

Pathway	Superclasses	Global FDR	p.value (0 - 6)	p.value (0 - 12)	p.value (6 - 12)
L.citrulline.biosynthesis	Amino.acid.biosynthesis	0.0000	large green	black	large red
Urea.cycle	Amino.acid.biosynthesis	0.0000	large green	black	large red
Calvin.Benson.Bassham.cycle	Carbohydrate.biosynthesis	0.0000	large red	black	large green
Pentose.phosphate.pathway (non.oxidative.branch)	Pentose.phosphate.pathways	0.0000	large red	black	large green
Glycolysis.II (fructose.6.phosphate)	Glycolysis	0.0000	large green	black	large red
Glycolysis I (glucose.6.phosphate)	Glycolysis	0.0000	large green	black	large red
Homolactic.fermentation	Fermentation	0.0000	large green	black	black
Gluconeogenesis.III	Carbohydrate.biosynthesis	0.0000	large green	black	large red
Glycogen.degradation.II (eukaryotic)	Carbohydrate.degradation	0.0001	medium red	black	large green
Glycolysis.IV (plant.cytosol)	Glycolysis	0.0039	medium green	black	medium red
Acetyl.CoA.fermentation.to.butanoate.II	Fermentation	0.0043	medium red	black	small green
Glycolysis.VI (metazoan)	Glycolysis	0.0094	medium green	black	small red
Superpathway.of.hexitol.degradation (bacteria)	Secondary.metabolite.degradation	0.0094	medium green	black	small red
Chorismate.biosynthesis.from.3.dehydroquinate	Aromatic.compound.biosynthesis	0.0118	medium red	black	small green
GDP.mannose.biosynthesis	Carbohydrate.biosynthesis	0.0118	medium red	black	black
Purine.nucleobases.degradation.I (anaerobic)	Nucleoside.Nucleotide.Degradation	0.0118	medium red	black	black
Superpathway.of.pyrimidine.ribonucleosides.degradation	Nucleoside.Nucleotide.Degradation	0.0118	medium green	black	small red
Tetrapyrrole.biosynthesis.I (from.glutamate)	Tetrapyrrole.biosynthesis	0.0118	medium red	black	black
L.rhamnose.degradation.I	Carbohydrate.degradation	0.0159	medium red	black	medium green
D.galactose.degradation.V (Leloir.pathway)	Carbohydrate.degradation	0.0174	medium red	black	small green
Dalactose.degradation.I (Leloir.pathway)	Carbohydrate.degradation	0.0174	medium red	black	small green
Guanosine.nucleotides.degradation.II	Nucleoside.Nucleotide.Degradation	0.0246	medium red	black	black
Chorismate.biosynthesis.I	Aromatic.compound.biosynthesis	0.0303	medium red	black	small green
Creatinine.degradation.I	Amine.polyamine.biosynthesis	0.0308	medium red	black	small green
L.methionine.biosynthesis.III	Amino.acid.biosynthesis	0.0325	medium red	black	small green
Palmitoleate.biosynthesis.I (from..5Z..dodec.5.enoate)	Fatty.acid.lipid.biosynthesis	0.0325	medium green	black	black
Superpathway.of.glycolysis.and.Entner.Doudoroff	Glycolysis	0.0325	small green	black	black
Superpathway.of.arginine.and.polyamine.biosynthesis	Amine.polyamine.biosynthesis	0.0332	small red	black	black
Gluconeogenesis.I	Carbohydrate.biosynthesis	0.0337	small green	black	black
Superpathway.of..beta..D.glucuronide.and.D.glucuronate.degradation	Carboxylate.degradation	0.0345	small red	black	black
Guanosine.nucleotides.degradation.III	Nucleoside.Nucleotide.Degradation	0.0347	small red	black	black
Superpathway.of.L.serine.and.glycine.biosynthesis.I	Amino.acid.biosynthesis	0.0357	small red	black	black
Superpathway.of.polyamine.biosynthesis.I	Amine.polyamine.biosynthesis	0.0380	small red	black	black
Superpathway.of.pyridoxal.5 (phosphate.biosynthesis.and.salvage)	Cofactor.Carrier.Vitamin.biosynthesis	0.0429	small green	black	small red
Adenosine.nucleotides.degradation.II	Nucleoside.Nucleotide.Degradation	0.0440	small red	black	small green
4.amino.2.methyl.5.phosphomethylpyrimidine.biosynthesis (yeast)	Cofactor.Carrier.Vitamin.biosynthesis	0.0487	small green	black	black
4.deoxy.L.threo.hex.4.enopyranuronate.degradation	Secondary.metabolite.degradation	0.0487	small red	black	small green
5.aminoimidazole.ribonucleotide.biosynthesis.I	Nucleoside.Nucleotide.biosynthesis	0.0487	small red	black	small green
5.aminoimidazole.ribonucleotide.biosynthesis.II	Nucleoside.Nucleotide.biosynthesis	0.0487	small red	black	small green
Biotin.biosynthesis.II	Cofactor.Carrier.Vitamin.biosynthesis	0.0487	small green	black	small red
Fatty.acid.elongation (saturated)	Fatty.acid.lipid.biosynthesis	0.0487	small green	black	small green
Flavin.biosynthesis.III	Cofactor.Carrier.Vitamin.biosynthesis	0.0487	small green	black	small red
Folate.transformations.II	Cofactor.Carrier.Vitamin.biosynthesis	0.0487	small green	black	black
N10.formyl.tetrahydrofolate.biosynthesis	Cofactor.Carrier.Vitamin.biosynthesis	0.0487	small green	black	small red
Palmitate.biosynthesis.II (bacteria.and.plants)	Fatty.acid.lipid.biosynthesis	0.0487	black	black	small green
Purine.ribonucleosides.degradation	Nucleoside.Nucleotide.Degradation	0.0487	black	black	small green

Table S2. Functional pathways encoded by the gut microbiota changed after taking dietary supplement. Table of the 46 individual pathways that significantly differed across timepoints. The metaCyc superclasses assigned to each pathway are shown. Significance determined by GEE comparisons. Circles represent pairwise adjusted *P* values of either increases or decreases in abundance determined by estimated marginal means. Red indicates decrease, green indicates increase and black indicates no significant change. Small circle adjusted *P* value between 0.01 - 0.05, medium circle adjusted *P* value between 0.001 and 0.01, large circle adjusted *P* value is < 0.001.

Extended Data Table 3: Upregulated KEGG pathway gene sets identified using thecamera function of limma following 6-weeks of HAMSAB supplementation

	**NGenes**	**Direction**	**PValue**	**FDR**
KEGG_PATHOGENIC_ESCHERICHIA_COLI_INFECTION	46	Up	1.03E-05	**0.00032561**
KEGG_SPLICEOSOME	123	Up	1.87E-05	**0.00051181**
KEGG_ENDOCYTOSIS	153	Up	2.09E-05	**0.00054291**
KEGG_RIBOSOME	85	Up	4.76E-05	**0.0009854**
KEGG_PARKINSONS_DISEASE	112	Up	0.00010368	**0.0016533**
KEGG_TOLL_LIKE_RECEPTOR_SIGNALING_PATHWAY	74	Up	0.00014285	**0.0020007**
KEGG_ALZHEIMERS_DISEASE	140	Up	0.00019282	**0.00243343**
**KEGG_OXIDATIVE_PHOSPHORYLATION**	114	Up	0.00022402	**0.00268366**
KEGG_MELANOMA	45	Up	0.00027273	**0.0030361**
KEGG_LEISHMANIA_INFECTION	62	Up	0.00029039	**0.00317275**
KEGG_ANTIGEN_PROCESSING_AND_PRESENTATION	59	Up	0.00033346	**0.00342159**
KEGG_EPITHELIAL_CELL_SIGNALING_IN_HELICOBACTER_PYLORI_INFECTION	55	Up	0.00070697	**0.00570295**
KEGG_HUNTINGTONS_DISEASE	152	Up	0.00076341	**0.00602555**
KEGG_NOD_LIKE_RECEPTOR_SIGNALING_PATHWAY	50	Up	0.00093818	**0.00686541**
KEGG_RENAL_CELL_CARCINOMA	60	Up	0.0010362	**0.00742162**
KEGG_PROTEASOME	41	Up	0.00109932	**0.0076194**
KEGG_BASAL_TRANSCRIPTION_FACTORS	27	Up	0.00126445	**0.00845356**
KEGG_CHEMOKINE_SIGNALING_PATHWAY	130	Up	0.00129201	**0.00858914**
KEGG_NEUROTROPHIN_SIGNALING_PATHWAY	105	Up	0.00144972	**0.00932547**
KEGG_REGULATION_OF_ACTIN_CYTOSKELETON	150	Up	0.00193589	**0.01149356**
KEGG_CELL_ADHESION_MOLECULES_CAMS	90	Up	0.00195375	**0.01157105**
KEGG_FOCAL_ADHESION	140	Up	0.00221472	**0.01282635**
KEGG_CARDIAC_MUSCLE_CONTRACTION	48	Up	0.00279329	**0.01533061**
KEGG_NATURAL_KILLER_CELL_MEDIATED_CYTOTOXICITY	94	Up	0.00291243	**0.01574646**

**KEGG_FATTY_ACID_METABOLISM**	31	Up	0.0029675	**0.01589851**
KEGG_BLADDER_CANCER	35	Up	0.00302643	**0.01615921**
KEGG_MAPK_SIGNALING_PATHWAY	188	Up	0.00360128	**0.01827748**
KEGG_GRAFT_VERSUS_HOST_DISEASE	29	Up	0.00372	**0.01873001**
KEGG_APOPTOSIS	78	Up	0.00388889	**0.01930131**
KEGG_RIG_I_LIKE_RECEPTOR_SIGNALING_PATHWAY	50	Up	0.00419738	**0.02048772**
KEGG_NUCLEOTIDE_EXCISION_REPAIR	43	Up	0.00450588	**0.02152606**
KEGG_P53_SIGNALING_PATHWAY	58	Up	0.00469188	**0.02214565**
KEGG_TYPE_I_DIABETES_MELLITUS	31	Up	0.0049238	**0.02287435**
KEGG_PANCREATIC_CANCER	60	Up	0.00505389	**0.02331806**
KEGG_GAP_JUNCTION	62	Up	0.00526513	**0.02405751**
KEGG_GLIOMA	52	Up	0.00576047	**0.02548211**
KEGG_UBIQUITIN_MEDIATED_PROTEOLYSIS	128	Up	0.00673348	**0.02852963**
KEGG_FC_GAMMA_R_MEDIATED_PHAGOCYTOSIS	82	Up	0.00742183	**0.03043531**
KEGG_PROSTATE_CANCER	78	Up	0.0075133	**0.03075697**
KEGG_LYSOSOME	114	Up	0.00767149	**0.03116128**
KEGG_LEUKOCYTE_TRANSENDOTHELIAL_MIGRATION	77	Up	0.00785845	**0.03158998**
KEGG_LONG_TERM_DEPRESSION	45	Up	0.00832359	**0.03282711**
KEGG_PATHWAYS_IN_CANCER	232	Up	0.00923581	**0.0354703**
KEGG_AUTOIMMUNE_THYROID_DISEASE	29	Up	0.00938201	**0.03582775**
KEGG_CYTOKINE_CYTOKINE_RECEPTOR_INTERACTION	142	Up	0.00948482	**0.03612752**
KEGG_O_GLYCAN_BIOSYNTHESIS	19	Up	0.01145614	**0.04146701**
KEGG_ALLOGRAFT_REJECTION	28	Up	0.01161694	**0.04174206**
KEGG_INSULIN_SIGNALING_PATHWAY	109	Up	0.01168677	**0.04190557**
KEGG_SYSTEMIC_LUPUS_ERYTHEMATOSUS	72	Up	0.0122219	**0.04305028**
KEGG_GLUTATHIONE_METABOLISM	37	Up	0.01305531	**0.04517673**
KEGG_ERBB_SIGNALING_PATHWAY	68	Up	0.01450521	**0.04835071**
KEGG_FC_EPSILON_RI_SIGNALING_PATHWAY	60	Up	0.01461368	**0.04854072**

KEGG_PEROXISOME	68	Up	0.01608822	0.05211834
KEGG_HYPERTROPHIC_CARDIOMYOPATHY_HCM	52	Up	0.01769088	0.05596578
KEGG_PRION_DISEASES	22	Up	0.01869297	0.05823816
KEGG_HISTIDINE_METABOLISM	17	Up	0.01985517	0.0607365
KEGG_ASTHMA	19	Up	0.02068978	0.06258573
KEGG_PANTOTHENATE_AND_COA_BIOSYNTHESIS	14	Up	0.02075427	0.06267445
KEGG_AMYOTROPHIC_LATERAL_SCLEROSIS_ALS	39	Up	0.03240687	0.08521562
KEGG_VIRAL_MYOCARDITIS	51	Up	0.03325894	0.08671254
KEGG_ADIPOCYTOKINE_SIGNALING_PATHWAY	52	Up	0.03549931	0.09071832
KEGG_CHRONIC_MYELOID_LEUKEMIA	65	Up	0.03662639	0.09294439
KEGG_ARRHYTHMOGENIC_RIGHT_VENTRICULAR_CARDIOMYOPATHY_ARVC	50	Up	0.03999511	0.09857803

Extended Table 4: Results of differential gene expression testing with EdgeR and RUVseq comparing baseline and week 6.

	Symbol	Kegg	Entrez	EnsId	Biotype	logFC	logCPM	LR	PValue	FDR
ENSG00000163293	NIPAL1	K02927;K156	152519	ENSG00000163293	protein_coding	0.89120734	-0.2292553	21.431591	3.67E-06	**0.02950508**
ENSG00000265830	AL592188.7	-	NA	NA	NA	-1.6461481	3.87234772	20.9804721	4.64E-06	**0.02950508**
ENSG00000151789	ZNF385D	-	79750	ENSG00000151789	protein_coding	0.97465004	-0.1851283	20.7996992	5.10E-06	**0.02950508**
ENSG00000256682	TAS2R12	-	NA	ENSG00000256682	transcribed_processed_pseu	-0.8103476	0.13631038	20.1731733	7.07E-06	**0.03069855**
ENSG00000244620	AL122127.25	-	NA	ENSG00000244620	lncRNA	-0.8226105	-0.2533075	17.9550127	2.26E-05	0.07852794
ENSG00000234737	KRT18P15	K07604	NA	ENSG00000234737	processed_pseudogene	-0.7636077	-0.0768158	16.3552774	5.25E-05	0.15191943
ENSG00000200176	RNU1-19P	-	NA	ENSG00000200176	snRNA	-1.7081984	-0.4483694	14.6018882	0.00013278	0.31017727
ENSG00000262372	RP11-669E14.6	-	NA	ENSG00000262372	lncRNA	-0.8576565	0.9211556	14.462905	0.00014295	0.31017727
ENSG00000242330	RN7SL683P	-	NA	ENSG00000242330	misc_RNA	-0.8904398	0.58026012	13.7810519	0.0002054	0.32443031
ENSG00000229531	RP1-102G20.5	-	NA	ENSG00000229531	lncRNA	-0.9716506	-0.6471222	13.6808892	0.00021665	0.32443031
ENSG00000243151	AL592188.1	-	NA	NA	NA	-1.4369439	0.8413489	13.6594133	0.00021914	0.32443031
ENSG00000213872	AC092798.2	K02912	NA	ENSG00000213872	processed_pseudogene	-0.5941114	1.27409491	13.2254338	0.00027618	0.32443031
ENSG00000232150	ST13P4	K09560	NA	ENSG00000232150	processed_pseudogene	0.79132674	-0.0039007	13.2098057	0.00027849	0.32443031
ENSG00000228939	AKT3-IT1	-	NA	ENSG00000228939	lncRNA	-1.0941103	0.49959936	13.0622369	0.00030131	0.32443031
ENSG00000230022	FNTAP2	K05955	NA	ENSG00000230022	processed_pseudogene	-0.6981356	0.2673223	12.9907356	0.00031304	0.32443031
ENSG00000179639	FCER1A	K08089	2205	ENSG00000179639	protein_coding	0.69640723	5.57304406	12.9068765	0.00032738	0.32443031
ENSG00000244926	ALKBH3-AS1	-	NA	ENSG00000244926	lncRNA	-0.6325209	0.8576022	12.6807062	0.00036945	0.32443031
ENSG00000227165	WDR11-AS1	-	NA	ENSG00000227165	lncRNA	0.64776202	1.82519829	12.6314678	0.00037931	0.32443031
ENSG00000184261	KCNK12	K04921	56660	ENSG00000184261	protein_coding	-0.7086483	0.60782211	12.4976605	0.00040746	0.32443031
ENSG00000213934	HBG1	K13824	3047	ENSG00000213934	protein_coding	3.89714598	3.43276172	12.3404407	0.00044325	0.32443031
ENSG00000259581	TYRO3P	K05116	NA	ENSG00000259581	processed_pseudogene	-0.9149902	0.74110044	12.3397649	0.00044341	0.32443031
ENSG00000199090	MIR326	-	NA	ENSG00000199090	miRNA	-1.2076769	-0.4754362	12.2335269	0.00046938	0.32443031
ENSG00000264827	AL592188.4	-	NA	NA	NA	-1.2460294	0.0559869	12.1870994	0.00048121	0.32443031
ENSG00000219712	RP11-532F6.2	-	NA	ENSG00000219712	processed_pseudogene	-0.8229917	0.60213829	12.1421356	0.00049295	0.32443031
ENSG00000236564	YWHAQP5	K01368	NA	ENSG00000236564	processed_pseudogene	-0.9190066	1.52792651	12.1135394	0.00050057	0.32443031
ENSG00000232545	KB-318B8.7	-	NA	ENSG00000232545	lncRNA	-0.8938741	0.80369888	12.0670249	0.00051322	0.32443031
ENSG00000246214	RP11-260E18.1	-	NA	ENSG00000246214	lncRNA	-0.8462127	-0.3832809	12.0199871	0.00052633	0.32443031
ENSG00000243423	RP5-837J1.1	K02922	NA	ENSG00000243423	processed_pseudogene	-0.7767684	-0.0455007	12.0045989	0.00053069	0.32443031
ENSG00000273245	RP11-434P11.2	-	NA	ENSG00000273245	lncRNA	-0.9898235	0.68240356	11.8614905	0.00057307	0.32443031
ENSG00000196565	HBG2	K13824	3048	ENSG00000196565	protein_coding	2.18279037	3.63887166	11.8047345	0.0005908	0.32443031
ENSG00000255139	AP000442.1	-	NA	ENSG00000255139	lncRNA	-0.7669982	-0.0717698	11.781517	0.00059822	0.32443031
ENSG00000233668	RP11-571F15.3	K02995	NA	ENSG00000233668	processed_pseudogene	-0.7889548	0.26123087	11.7635972	0.000604	0.32443031
ENSG00000231579	RPL7P21	-	NA	ENSG00000231579	processed_pseudogene	-0.7188238	0.68129816	11.7247235	0.00061675	0.32443031
ENSG00000169313	P2RY12	K04298	64805	ENSG00000169313	protein_coding	0.66653542	3.47099239	11.5206187	0.00068828	0.35140987
ENSG00000169224	GCSAML	-	148823	ENSG00000169224	protein_coding	0.7473603	1.90500889	11.2952994	0.00077704	0.37209139
ENSG00000114698	PLSCR4	-	57088	ENSG00000114698	protein_coding	0.8124095	0.3216348	11.2544785	0.00079431	0.37209139
ENSG00000122872	ARL4P	K07945	NA	ENSG00000122872	processed_pseudogene	-0.878271	0.01827066	11.2164842	0.00081074	0.37209139
ENSG00000226394	RP11-413E1.2	K03948	NA	ENSG00000226394	processed_pseudogene	-0.7065362	1.06315787	11.188214	0.00082319	0.37209139
ENSG00000226822	RP11-356N1.2	-	NA	ENSG00000226822	lncRNA	-0.7504548	0.14859655	11.159626	0.00083597	0.37209139
ENSG00000150681	RGS18	K16449	64407	ENSG00000150681	protein_coding	0.57056757	7.82878097	11.110042	0.00085862	0.37261766
ENSG00000267243	AC005307.3	-	NA	ENSG00000267243	lncRNA	0.78132256	-0.2084932	11.0241719	0.00089931	0.37650406
ENSG00000122043	LINC00544	-	NA	ENSG00000122043	lncRNA	-0.6086489	0.24314712	10.9820301	0.00092	0.37650406
ENSG00000222078	RN7SKP110	-	NA	ENSG00000222078	misc_RNA	-0.8642628	0.58909339	10.9567377	0.00093264	0.37650406

ENSG00000262803	RP11-160A9.3	-	NA	ENSG00000262803	processed_pseudogene	-0.6035765	0.59618593	10.7980811	0.00101605	0.39222015
ENSG00000269999	CTD-3185P2.2	-	NA	NA	NA	-0.6058098	0.06204701	10.7967977	0.00101676	0.39222015
ENSG00000242100	RPL9P32	K02940	NA	ENSG00000242100	processed_pseudogene	-1.1882256	0.3362082	10.6410903	0.00110602	0.39833901
ENSG00000135525	MAP7	K10433	9053	ENSG00000135525	protein_coding	0.5855958	2.34012891	10.6205574	0.00111837	0.39833901
ENSG00000249693	THEGL	-	100506564	ENSG00000249693	protein_coding	0.66788795	-0.0567775	10.6137368	0.00112251	0.39833901
ENSG00000270127	RP11-526I2.5	-	NA	ENSG00000270127	lncRNA	-0.5692056	2.45076263	10.6106053	0.00112441	0.39833901
ENSG00000140287	HDC	K01590	3067	ENSG00000140287	protein_coding	0.66616549	3.65920714	10.4632352	0.00121774	0.41460711
ENSG00000236035	RP11-90O23.1	K14842	NA	ENSG00000236035	processed_pseudogene	-0.8446971	0.32008075	10.4626896	0.0012181	0.41460711
ENSG00000168350	DEGS2	K04712	123099	ENSG00000168350	protein_coding	-0.5900463	-0.228832	10.362431	0.00128606	0.42152871
ENSG00000267529	RP11-53B2.1	-	NA	ENSG00000267529	lncRNA	-0.5802076	1.44944206	10.3462963	0.00129735	0.42152871
ENSG00000149534	MS4A2	K08090	2206	ENSG00000149534	protein_coding	0.65017913	1.62677239	10.3265761	0.00131128	0.42152871
ENSG00000090104	RGS1	K16449	5996	ENSG00000090104	protein_coding	0.6924649	0.92880024	10.2270516	0.00138396	0.43542912
ENSG00000257802	MRS2P2	K16075	NA	ENSG00000257802	processed_pseudogene	-0.5811773	1.39896949	10.1950498	0.00140818	0.43542912
ENSG00000250030	RP11-584P21.4	K12581	NA	ENSG00000250030	processed_pseudogene	-0.7250826	-0.2792747	10.0720188	0.00150538	0.43542912
ENSG00000178033	FAM26E	-	254228	ENSG00000178033	protein_coding	0.69042334	0.08536618	10.0586885	0.00151631	0.43542912
ENSG00000235740	RP11-436I24.1	-	NA	ENSG00000235740	lncRNA	-0.7618186	2.49456478	10.0552841	0.00151912	0.43542912
ENSG00000180346	TIGD2	-	166815	ENSG00000180346	protein_coding	0.38947263	2.11125121	9.93964099	0.00161757	0.43542912
ENSG00000121769	FABP3	K08752	2170	ENSG00000121769	protein_coding	-0.6654635	0.64344034	9.80970354	0.00173593	0.43542912
ENSG00000272412	Metazoa_SRP	-	NA	ENSG00000272412	misc_RNA	-0.9478426	-0.7710013	9.78658443	0.0017579	0.43542912
ENSG00000133477	FAM83F	K12581;K029	113828	ENSG00000133477	protein_coding	0.74554827	0.32466309	9.78568989	0.00175875	0.43542912
ENSG00000146122	DAAM2	K04512	23500	ENSG00000146122	protein_coding	0.75304352	1.1087293	9.75941283	0.00178407	0.43542912
ENSG00000242609	RP11-398A8.1	-	NA	ENSG00000242609	processed_pseudogene	-0.943753	-0.2999307	9.70985606	0.00183282	0.43542912
ENSG00000244556	ODCP	K01581	NA	ENSG00000244556	processed_pseudogene	-0.6129997	1.39159499	9.67699441	0.0018659	0.43542912
ENSG00000270772	RP11-332H21.2	K00161	NA	ENSG00000270772	processed_pseudogene	-0.7537208	0.75104364	9.66135551	0.00188185	0.43542912
ENSG00000244071	RPL9P33	-	NA	ENSG00000244071	processed_pseudogene	-0.9680644	0.55139976	9.61127797	0.00193386	0.43542912
ENSG00000148498	PARD3	K04237	56288	ENSG00000148498	protein_coding	0.62730521	2.39204056	9.58654404	0.00196009	0.43542912
ENSG00000214283	RP11-85F14.1	-	NA	ENSG00000214283	processed_pseudogene	-0.7851905	0.50127068	9.55691682	0.00199197	0.43542912
ENSG00000225213	RP11-197M22.2	-	NA	ENSG00000225213	lncRNA	-0.9126482	0.00328871	9.50683701	0.00204708	0.43542912
ENSG00000224072	RP11-75A9.3	-	NA	ENSG00000224072	processed_pseudogene	-0.5018623	1.21348529	9.47028445	0.00208827	0.43542912
ENSG00000200156	RNU5B-1	-	NA	ENSG00000200156	snRNA	-1.9344189	3.05544087	9.46234796	0.00209732	0.43542912
ENSG00000160193	WDR4	K15443	10785	ENSG00000160193	protein_coding	-0.6018569	2.55334198	9.44819244	0.00211357	0.43542912
ENSG00000238961	SNORA47	-	NA	ENSG00000238961	snoRNA	0.54676923	1.63270152	9.41221088	0.00215545	0.43542912
ENSG00000143429	AC027612.6	K14957	NA	ENSG00000143429	transcribed_unprocessed_ps	0.63456364	-0.0235772	9.40603674	0.00216272	0.43542912
ENSG00000266222	RP11-433M22.1	-	NA	ENSG00000266222	lncRNA	-1.3786895	-0.5765254	9.39986868	0.00217001	0.43542912
ENSG00000147650	LRP12	K12581;K092	29967	ENSG00000147650	protein_coding	0.46231259	2.05756168	9.39796876	0.00217226	0.43542912
ENSG00000264878	Z85986.1	-	NA	NA	NA	-0.8204803	0.64166997	9.39637173	0.00217415	0.43542912
ENSG00000105971	CAV2	K12958	858	ENSG00000105971	protein_coding	0.89624386	0.56507987	9.39170381	0.0021797	0.43542912
ENSG00000169902	TPST1	K01021	8460	ENSG00000169902	protein_coding	0.63024029	3.76623831	9.36097355	0.00221654	0.43542912
ENSG00000259165	DDX18P1	K13179	NA	ENSG00000259165	processed_pseudogene	-0.4794796	2.10319687	9.26691834	0.0023333	0.43542912
ENSG00000213216	RP11-355O1.7	K02975	NA	ENSG00000213216	processed_pseudogene	-0.5374137	-0.3541348	9.23924616	0.00236882	0.43542912
ENSG00000134317	GRHL1	K09275	29841	ENSG00000134317	protein_coding	0.7113523	1.50511366	9.22652942	0.00238533	0.43542912
ENSG00000180822	PSMG4	K11878;K114	389362	ENSG00000180822	protein_coding	-0.5626576	4.03413138	9.20162623	0.002418	0.43542912
ENSG00000261096	RP11-690I21.2	-	NA	ENSG00000261096	lncRNA	-0.9275259	0.29512228	9.19237684	0.00243025	0.43542912
ENSG00000259781	RP11-673C5.1	K10802	NA	ENSG00000259781	processed_pseudogene	0.55768482	2.01003051	9.1814456	0.00244481	0.43542912
ENSG00000258839	MC1R	K04199	4157	ENSG00000258839	protein_coding	-0.4984494	1.88646697	9.14375169	0.00249569	0.43542912
ENSG00000241230	RN7SL801P	-	NA	ENSG00000241230	misc_RNA	-0.9994051	-0.8719354	9.13207818	0.00251166	0.43542912

ENSG00000225084	AL450226.2	-	NA	ENSG00000225084	lncRNA	-1.0944883	-0.6367515	9.12872184	0.00251627	0.43542912
ENSG00000233387	RP11-342D11.3	-	NA	ENSG00000233387	lncRNA	-0.5962417	1.01611416	9.11643451	0.00253323	0.43542912
ENSG00000218313	RP11-393I2.2	K03004	NA	ENSG00000218313	processed_pseudogene	-0.7196903	2.27709968	9.10218276	0.00255305	0.43542912
ENSG00000206172	HBA1	K13822	3039	ENSG00000206172	protein_coding	2.57425627	6.79071081	9.1016418	0.0025538	0.43542912
ENSG00000252949	AL136303.1	-	NA	NA	NA	-0.7734006	-0.0439744	9.09317747	0.00256565	0.43542912
ENSG00000186049	KRT73	K07605	319101	ENSG00000186049	protein_coding	-0.6310341	1.3030057	9.05516224	0.00261954	0.43542912
ENSG00000137331	IER3	-	8870	ENSG00000137331	protein_coding	0.57824846	2.48659946	9.03098965	0.00265441	0.43542912
ENSG00000270711	RP11-46A10.8	-	NA	ENSG00000270711	processed_pseudogene	-0.5642782	-0.096552	9.02601523	0.00266164	0.43542912
ENSG00000259073	FOXN3-AS2	-	NA	ENSG00000259073	lncRNA	-0.9905673	1.93736594	9.01068733	0.00268405	0.43542912
ENSG00000198829	SUCNR1	K10042	56670	ENSG00000198829	protein_coding	0.69733164	1.00221041	8.98718661	0.00271879	0.43542912
ENSG00000233337	UBE2FP3	K10687	NA	ENSG00000233337	processed_pseudogene	-0.555005	0.63784693	8.97256292	0.00274064	0.43542912
ENSG00000175105	ZNF654	-	55279	ENSG00000175105	protein_coding	0.41828049	5.20917483	8.96892697	0.0027461	0.43542912
ENSG00000260536	RP5-1085F17.4	-	NA	ENSG00000260536	lncRNA	-0.6386072	-0.0867436	8.95953932	0.00276024	0.43542912
ENSG00000225338	RP11-384C4.3	K02893	NA	ENSG00000225338	processed_pseudogene	-0.7379865	0.9478958	8.94833201	0.00277723	0.43542912
ENSG00000225135	RP11-361F15.2	-	NA	NA	NA	0.57893038	3.79132538	8.92457141	0.00281359	0.43542912
ENSG00000248477	RP11-848G14.2	K12807	NA	ENSG00000248477	transcribed_unprocessed_ps	-0.601899	-0.0471494	8.9220552	0.00281747	0.43542912
ENSG00000041353	RAB27B	K07886	5874	ENSG00000041353	protein_coding	0.53812147	6.06263225	8.92148931	0.00281835	0.43542912
ENSG00000251194	RP1-68D18.2	-	NA	ENSG00000251194	lncRNA	-0.4370391	2.39129262	8.91889817	0.00282235	0.43542912
ENSG00000254556	AF131215.4	-	NA	ENSG00000254556	lncRNA	-0.9212989	1.6966818	8.91199385	0.00283304	0.43542912
ENSG00000236086	HMGN2P28	K11300	NA	ENSG00000236086	processed_pseudogene	-0.5542456	-0.1943794	8.89532003	0.00285902	0.43542912
ENSG00000116918	TSNAX	K17985	7257	ENSG00000116918	protein_coding	0.39627946	5.54421015	8.89500285	0.00285952	0.43542912
ENSG00000154188	ANGPT1	K05465	284	ENSG00000154188	protein_coding	0.6714805	3.10272011	8.85886152	0.00291669	0.43542912
ENSG00000259274	CTD-2501E16.2	-	NA	ENSG00000259274	lncRNA	-0.5521034	0.60660058	8.81787581	0.00298294	0.43542912
ENSG00000151023	ENKUR	-	219670	ENSG00000151023	protein_coding	0.59257685	2.70603105	8.80094614	0.00301074	0.43542912
ENSG00000237672	KRR1P1	K06961	NA	ENSG00000237672	processed_pseudogene	-0.571237	1.24881057	8.79756975	0.00301632	0.43542912
ENSG00000268362	CTD-2017D11.1	-	NA	ENSG00000268362	lncRNA	-0.6276071	3.35680106	8.76225313	0.00307529	0.43542912
ENSG00000272486	RP11-532M24.1	-	NA	NA	NA	-0.5946875	0.33231092	8.75490148	0.00308771	0.43542912
ENSG00000119862	LGALSL	-	29094	ENSG00000119862	protein_coding	0.46801104	4.56850063	8.73132177	0.0031279	0.43542912
ENSG00000181227	RP4-682C21.2	K00658	NA	ENSG00000181227	processed_pseudogene	-0.9470581	0.49666119	8.72546921	0.00313795	0.43542912
ENSG00000231697	NANOGP5	K10164	NA	ENSG00000231697	transcribed_processed_pseu	-0.6068257	0.17112729	8.71599316	0.00315431	0.43542912
ENSG00000255320	RP11-755F10.1	-	NA	ENSG00000255320	lncRNA	-1.6680829	-0.3842963	8.70020766	0.00318174	0.43542912
ENSG00000234500	GS1-124K5.10	K03121	NA	ENSG00000234500	unprocessed_pseudogene	-0.9128438	-0.3655969	8.68528377	0.0032079	0.43542912
ENSG00000270130	RP11-214K3.23	-	NA	ENSG00000270130	lncRNA	-0.5547594	0.79447858	8.65940702	0.00325377	0.43542912
ENSG00000258485	SRMP2	K00797	NA	ENSG00000258485	processed_pseudogene	-0.9512417	-0.5645284	8.65477867	0.00326205	0.43542912
ENSG00000261574	RP1-168P16.2	-	NA	NA	NA	-1.1349624	1.4911027	8.63871187	0.00329094	0.43542912
ENSG00000247473	CARS-AS1	-	NA	ENSG00000247473	lncRNA	-0.5126052	0.60729991	8.62761217	0.00331105	0.43542912
ENSG00000230581	RP11-390F4.8	K05692	NA	ENSG00000230581	processed_pseudogene	-0.6422787	0.31945708	8.62044233	0.00332411	0.43542912
ENSG00000168497	SDPR	K19387	8436	ENSG00000168497	protein_coding	0.49879442	7.23502216	8.61918539	0.00332641	0.43542912
ENSG00000250159	RP11-381K20.2	-	NA	ENSG00000250159	lncRNA	-0.5343155	0.5247808	8.58220618	0.00339464	0.43542912
ENSG00000229728	RP11-314N13.3	-	NA	ENSG00000229728	lncRNA	-0.5247487	0.8405925	8.57838776	0.00340176	0.43542912
ENSG00000245534	RP11-219B17.1	-	NA	ENSG00000245534	lncRNA	-0.4744596	2.65463068	8.55909456	0.003438	0.43542912
ENSG00000236576	RP11-22B10.3	-	NA	ENSG00000236576	processed_pseudogene	-0.7223543	1.02486773	8.53033172	0.00349276	0.43542912
ENSG00000244229	RPL26P35	K02898	NA	ENSG00000244229	processed_pseudogene	-0.6495786	-0.4590881	8.50185917	0.00354784	0.43542912
ENSG00000119698	PPP4R4	K15426	57718	ENSG00000119698	protein_coding	0.6224526	0.11776759	8.47790367	0.00359486	0.43542912
ENSG00000225300	RP11-439E19.1	-	NA	ENSG00000225300	lncRNA	-0.7284191	1.2147235	8.4591367	0.00363214	0.43542912
ENSG00000250903	GMDS-AS1	-	NA	ENSG00000250903	lncRNA	-0.3849423	4.2375539	8.44486094	0.00366076	0.43542912

ENSG00000178538	CA8	K01672	767	ENSG00000178538	protein_coding	0.58018021	1.72011573	8.44411366	0.00366227	0.43542912
ENSG00000226705	SDCBPP1	K17254	NA	ENSG00000226705	processed_pseudogene	-1.0848284	-0.1764493	8.41923382	0.00371272	0.43542912
ENSG00000243637	AC019221.4	-	NA	NA	NA	-0.706938	-0.1086778	8.41839186	0.00371444	0.43542912
ENSG00000272498	RP11-415F23.3	-	NA	ENSG00000272498	lncRNA	-0.5427869	2.80750313	8.40167931	0.00374875	0.43542912
ENSG00000230124	RP5-1180C10.2	-	84320	ENSG00000230124	protein_coding	-0.6910051	1.97814773	8.39954306	0.00375315	0.43542912
ENSG00000122025	FLT3	K05092	2322	ENSG00000122025	protein_coding	0.59233982	3.12378094	8.37554554	0.00380303	0.43542912
ENSG00000267694	RP11-691H4.4	-	NA	ENSG00000267694	lncRNA	-0.765662	0.72664129	8.37464987	0.00380491	0.43542912
ENSG00000261451	RP11-981G7.1	-	NA	ENSG00000261451	lncRNA	-0.5503137	1.75711406	8.3722053	0.00381003	0.43542912
ENSG00000260711	RP11-747H7.3	-	NA	ENSG00000260711	lncRNA	-0.6416419	3.2541186	8.35733955	0.00384133	0.43542912
ENSG00000188536	HBA2	K13822	3040	ENSG00000188536	protein_coding	2.46026789	8.58469488	8.32661597	0.00390684	0.43542912
ENSG00000264630	RP11-4F22.2	-	NA	ENSG00000264630	lncRNA	-1.164038	1.70746362	8.32258751	0.00391551	0.43542912
ENSG00000185899	TAS2R60	K08474	338398	ENSG00000185899	protein_coding	-0.9513838	-0.3185196	8.2937732	0.00397812	0.43542912
ENSG00000254254	RP11-17A4.2	-	NA	ENSG00000254254	lncRNA	-0.5536185	-0.1655095	8.28134963	0.00400543	0.43542912
ENSG00000264462	MIR3648	-	NA	ENSG00000264462	miRNA	-2.4334551	4.84918522	8.26822478	0.00403449	0.43542912
ENSG00000227189	AC092535.3	-	NA	ENSG00000227189	lncRNA	-1.1436806	-0.456219	8.26575249	0.00403999	0.43542912
ENSG00000242716	RNA5-8S5	-	NA	NA	NA	-1.307075	1.89357817	8.22893622	0.00412277	0.43542912
ENSG00000235398	LINC00623	-	NA	NA	NA	0.48280967	-0.0651094	8.22517215	0.00413133	0.43542912
ENSG00000261308	RP11-923I11.7	-	401720	ENSG00000261308	protein_coding	-0.4544419	0.92082726	8.21613505	0.00415195	0.43542912
ENSG00000169439	SDC2	K16336	6383	ENSG00000169439	protein_coding	0.56513838	0.65496335	8.20103147	0.00418666	0.43542912
ENSG00000240356	RPL23AP7	K02893	NA	ENSG00000240356	transcribed_processed_pseu	0.53256584	2.75263848	8.18689088	0.00421942	0.43542912
ENSG00000254879	AC103828.1	-	NA	ENSG00000254879	lncRNA	-0.8749204	1.26048295	8.1857995	0.00422196	0.43542912
ENSG00000170011	MYRIP	-	25924	ENSG00000170011	protein_coding	-0.5560325	1.66232411	8.18453199	0.00422491	0.43542912
ENSG00000102554	KLF5	K09206	688	ENSG00000102554	protein_coding	0.49237617	1.96774472	8.17622084	0.00424431	0.43542912
ENSG00000229052	RP11-386I23.1	K17985	NA	ENSG00000229052	transcribed_processed_pseu	-0.7306023	1.64187799	8.16790808	0.00426381	0.43542912
ENSG00000207129	RNA5SP187	-	NA	ENSG00000207129	rRNA_pseudogene	-0.4863946	0.63316606	8.16262206	0.00427625	0.43542912
ENSG00000267702	RP11-53B2.2	-	NA	ENSG00000267702	lncRNA	-0.6823952	4.10014604	8.15986722	0.00428275	0.43542912
ENSG00000184825	HIST1H2AH	K11251	NA	NA	NA	-0.5869907	2.80966761	8.15764436	0.004288	0.43542912
ENSG00000240935	PLGLA	K01315	NA	ENSG00000240935	transcribed_unprocessed_ps	-0.8201849	-0.5013883	8.1532354	0.00429844	0.43542912
ENSG00000244180	AL592188.2	-	NA	NA	NA	-1.8235646	4.69288952	8.15232181	0.00430061	0.43542912
ENSG00000100678	SLC8A3	K05849	6547	ENSG00000100678	protein_coding	0.64520648	0.25098556	8.14678766	0.00431375	0.43542912
ENSG00000232564	RP4-591N18.2	-	NA	ENSG00000232564	lncRNA	-0.7202825	1.48679691	8.14370444	0.00432109	0.43542912
ENSG00000128536	CDHR3	K16503;K114	222256	ENSG00000128536	protein_coding	-0.5386647	2.39040826	8.141044	0.00432744	0.43542912
ENSG00000196312	HIATL2	-	84278	ENSG00000196312	transcribed_unprocessed_ps	-0.3646159	2.9859268	8.13609868	0.00433925	0.43542912
ENSG00000262074	SNORD3B-2	-	NA	ENSG00000262074	snoRNA	-1.1217256	0.40683584	8.12100494	0.00437553	0.43542912
ENSG00000226803	RP11-203B9.4	-	NA	ENSG00000226803	lncRNA	-0.4090472	2.54123601	8.10966933	0.00440298	0.43542912
ENSG00000244734	HBB	K13823	3043	ENSG00000244734	protein_coding	3.30244583	9.6144834	8.10308024	0.00441901	0.43542912
ENSG00000105982	RNF32	-	140545	ENSG00000105982	protein_coding	-0.4066835	1.76876839	8.09402861	0.00444113	0.43542912
ENSG00000200397	Y_RNA	-	NA	ENSG00000200397	misc_RNA	-0.4431899	0.39675556	8.0743391	0.00448965	0.43542912
ENSG00000254481	PTP4A2P2	K18041	NA	ENSG00000254481	processed_pseudogene	0.50255239	0.90021554	8.06518284	0.00451239	0.43542912
ENSG00000268367	AC126614.1	K02927	NA	NA	NA	-0.9876112	-0.5321461	8.06411322	0.00451506	0.43542912
ENSG00000228492	RAB11FIP1P1	K12484	NA	ENSG00000228492	processed_pseudogene	-0.5886651	2.45479362	8.05718896	0.00453234	0.43542912
ENSG00000223899	SEC13P1	K14004	NA	ENSG00000223899	processed_pseudogene	-0.7329607	-0.3933527	8.05228604	0.00454463	0.43542912
ENSG00000266378	RP11-214O1.3	-	NA	ENSG00000266378	lncRNA	-1.0299226	0.80746186	8.04219452	0.00457001	0.43542912

Mass cytometry panel used for immunophenotyping

**Metal Isotope /Fluorochrome**	**Antigen**	**Clone**	**Manufacturer**
Barcoding and fluorescent			
^{104}Pd, ^{106}Pd or ^{108}Pd	CD45	30-F11	BD/Biolegend
Biotin	CD86	IT2.2	BD
AF647	CD160	BY55	BD
Remaining surface stain			
^{89}Y	CD11c	Bu15	Biolegend
^{113}In	IgM	G20127	BD
^{115}In	Biotin	1D4-C5	Biolegend
^{139}La	CD56	NCAM16.2	BD
^{141}Pr	CD27	M-T271	BD
^{142}Nd	CD19	HIB19	Biolegend
^{143}Nd	CD45RA	HI100	BD
^{144}Nd	TCRgd	B1	Biolegend
^{145}Nd	CD4	RPA-T4	Biolegend
^{146}Nd	IgD	IA6-2	BD
^{147}Sm	Cy5 (for AF647)	CY5-15	Sigma
^{149}Sm	CD366 (Tim3)	7D3	BD
^{150}Nd	KLRG1	SA231A	Biolegend
^{151}Eu	CD123	6H6	Biolegend
^{152}Sm	CD45RO	UCHL1	Biolegend
^{153}Eu	CD62L	DREG	Biolegend
^{154}Gd	CD3	UCHT1	BD
^{156}Gd	CD279 (PD-1)	EH12 .2H7	Biolegend
^{158}Gd	CD87	VIM5	Biolegend
^{159}Tb	CD223 (Lag3)	17B4	Genetex
^{161}Dy	CD274 (PD-L1)	MIH1	BD
^{165}Ho	CD16	3G8	BD
^{166}Er	TIGIT	MBSA43	eBioscience
^{167}Er	CD66a	YTH71.3	Abcam
^{168}Er	CD8a	RPA-T8	BD
^{169}Tm	CD25	M-A251	Biolegend
^{170}Er	CD152 (CTLA4)	14D3	eBioscience
^{172}Yb	CD197 (CCR7)	G043H7	Biolegend

**Metal Isotope /Fluorochrome**	**Antigen**	**Clone**	**Manufacturer**
Intracellular stain			
^{148}Nd	Helios	22F6	Nobus Bio
^{155}Gd	EOMES	WD1928	eBioscience
^{162}Er	FOXP3	PCH101	eBioscience
^{163}Dy	BLIMP	6D3	BD
^{164}Er	GATA3	L50-823	BD
^{171}Yb	GranzymeB	REA226	Miltenyi
^{175}Lu	Perforin	dG9	Biolegend
$^{191/193}Ir$	DNA Intercalator	-	Fluidigm
^{209}Bi	T-bet	4B10	BD
